# Steerable and Agile Light‐Fueled Rolling Locomotors by Curvature‐Engineered Torsional Torque

**DOI:** 10.1002/advs.202304715

**Published:** 2023-08-11

**Authors:** Jun‐Chan Choi, Jisoo Jeon, Jae‐Won Lee, Asad Nauman, Jae Gyeong Lee, Woongbi Cho, Chanwoo Lee, Young‐Min Cho, Jeong Jae Wie, Hak‐Rin Kim

**Affiliations:** ^1^ School of Electronic and Electrical Engineering Kyungpook National University 41566 Daegu Republic of Korea; ^2^ Soft Hybrid Materials Research Center Korea Institute of Science and Technology 02792 Seoul Republic of Korea; ^3^ Program in Environmental and Polymer Engineering Inha University 22212 Incheon Republic of Korea; ^4^ Department of Organic and Nano Engineering Hanyang University 04763 Seoul Republic of Korea; ^5^ Human‐Tech Convergence Program Hanyang University 04763 Seoul Republic of Korea; ^6^ School of Electronics Engineering Kyungpook National University 41566 Daegu Republic of Korea; ^7^ Department of Chemical Engineering Hanyang University 04763 Seoul Republic of Korea; ^8^ Institute of Nano Science and Technology Hanyang University 04763 Seoul Republic of Korea; ^9^ The Research Institute of Industrial Science Hanyang University Seoul 04763 Republic of Korea; ^10^ The Michael M. Szwarc Polymer Research Institute State University of New York College of Environmental Science and Forestry Syracuse NY 13210 USA; ^11^ Department of Chemical Engineering State University of New York College of Enviromental Science and Forestry Syracuse NY 13210 USA

**Keywords:** amphibious multimodal actuation, helical soft robot, liquid crystal polymer network, on‐demand steering, photo‐mechanical rolling

## Abstract

On‐demand photo‐steerable amphibious rolling motions are generated by the structural engineering of monolithic soft locomotors. Photo‐morphogenesis of azobenzene‐functionalized liquid crystal polymer networks (azo‐LCNs) is designed from spiral ribbon to helicoid helices, employing a 270° super‐twisted nematic molecular geometry with aspect ratio variations of azo‐LCN strips. Unlike the intermittent and biased rolling of spiral ribbon azo‐LCNs with center‐of‐mass shifting, the axial torsional torque of helicoid azo‐LCNs enables continuous and straight rolling at high rotation rates (≈720 rpm). Furthermore, center‐tapered helicoid structures with wide edges are introduced for effectively accelerating photo‐motilities while maintaining directional controllability. Irrespective of surface conditions, the photo‐induced rotational torque of center‐tapered helicoid azo‐LCNs can be transferred to interacting surfaces, as manifested by steep slope climbing and paddle‐like swimming multimodal motilities. Finally, the authors demonstrate continuous curvilinear guidance of soft locomotors, bypassing obstacles and reaching desired destinations through real‐time on‐demand photo‐steering.

## Graphical abstract

The macroscopic helical structure of the monolithic rolling locomotor is structurally engineered using molecular machinery embedded liquid crystal polymer strips with 270° super‐twisted nematic alignment. Center‐tapered helicoid locomotor exhibits agile and steerable photo‐motilities via axial rotation and deformation. This allows amphibious rolling, such as climbing slopes or swimming in liquids, and bypassing obstacles to reach the desired destination.

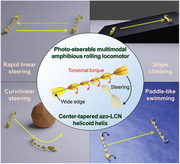

## 1 Introduction

The rolling wheel, which transduces axial rotation into linear movement, has been regarded as one of the most historically significant human inventions in providing energy‐efficient and fast‐moving or carrying methods.^[^ ^1^, ^2^ ^]^ In nature, various living creatures also adopt a rolling motion to quickly escape dangerous situations from predators. For example, the Pleurotya caterpillar anchors its tail to the ground and bends its body into a wheel shape to perform a rolling motion tens of times faster than its crawling velocity.^[^ ^3^, ^4^ ^]^ Owing to agile motility, the rolling mode has been researched in various untethered soft robots by repeating the reversible stimuli‐responsive deformations of soft bodies. To determine the rolling direction, rolling torque is generated by breaking circular symmetry at the cross‐section perpendicular to the rotational axis.^[^ ^5^, ^6^, ^7^, ^8^, ^9^, ^10^, ^11^, ^12^, ^13^, ^14^ ^]^ In general, rolling soft robots adopt symmetric geometries, such as rod‐like,^[^ ^7^, ^8^ ^]^ cylinder‐like,^[^ ^9^, ^10^, ^11^ ^]^ or spring‐like structures,^[^ ^12^, ^13^, ^14^ ^]^ for agile motilities. However, the geometries of the rolling soft robots mentioned above make it difficult to control the direction of movement because the rolling axis remains straight without axial deformation, which eclipses the benefits of agile motility. To implement both agile and steerable rolling motions, structural engineering of soft robots is required for macroscopic asymmetric axial bending deformations of the rolling axis, along with a fast‐rotational rate.

In developing the modal types and modal efficiencies of stimuli‐responsive untethered soft robots via the design of shape morphing, liquid crystalline polymers (LCPs) provide viable ways to predesign molecular architectures and resultant macroscopic modal actuation under external stimuli. The spatially patternable long‐range ordering effects of LCPs and their anisotropic stress distributions can be controlled for modal actuation through stimuli‐dependent directional LCP contraction or swelling by applying various external stimuli including heat,^[^ ^15^, ^16^ ^]^ humidity,^[^ ^17^, ^18^ ^]^ magnetic fields,^[^ ^19^, ^20^, ^21^ ^]^ and light.^[^ ^22^, ^23^, ^24^, ^25^, ^26^, ^27^, ^28^, ^29^, ^30^ ^]^ In particular, the photo‐mechanical actuation of LCPs has advantages in diversifying contactless actuation modalities for miniature and lightweight soft robots, thus allowing the realization of various biomimetic motions such as crawling,^[^ ^22^, ^23^, ^24^ ^]^ swimming,^[^ ^25^ ^]^ jumping,^[^ ^26^ ^]^ and rolling^[^ ^27^, ^28^, ^29^, ^30^ ^]^ in monolithic structures. By employing facile spatiotemporal control of the beam flux patterns, light‐responsive LCPs can achieve sequential real‐time control. In the case of photo‐mediated rolling soft robots, moving direction control between forward and backward rolling can be selected by operating the biased actinic light flux to break the cross‐sectional circular symmetry of the rolling axis.^[^ ^27^, ^28^, ^29^ ^]^ Research on rolling controls with directional turning, which requires geometrical asymmetry along the rolling axis, has been limited to date. Recently, a light‐guided omnidirectional rolling scheme was reported in a soft robot that employs photo‐responsive Kirigami bundles at edges, even though the rolling motility of its moving speed (evaluated by scaling its rolling body) was slower than that of soft robots with unidirectional controllability.^[^ ^30^ ^]^ In addition, most previously reported rolling motilities showed unstable intermittent rolling motions ^[^ ^28^, ^29^ ^]^ arising from temporal competitive or synergetic relationships between a shifting center of mass in the rolling body and cross‐sectional geometric shape deformation, which also degrades rolling motilities of photo‐fueled rolling speed and photo‐guided rolling directionality.

Here, we report the structural engineering of a monolithic soft locomotor for rapid, yet steerable, photo‐fueled rolling powered by helicoidal torsional torque. An azobenzene‐functionalized liquid crystal polymer network (azo‐LCN) strip is photo‐structured into helical structures to improve rolling dynamics. Through the molecular alignment design in the in‐plane and depth directions of the azo‐LCN strip, we systematically explored the effects of macroscopic curvature with helix formation by photo‐structuring and the resultant photo‐fueled rolling mechanisms. The length‐to‐width aspect ratio (AR) of the azo‐LCN strip was strictly regulated by laser cutting to investigate different shape morphing of the helix, varying from the spiral ribbon to the helicoid structures. Upon exposure to actinic light, low AR azo‐LCN films were shape‐morphed into spiral ribbons by out‐of‐plane bending along the central line of the helix, generating relatively large rolling diameters. Since the spiral ribbon has a biased contact interface with the surface, the rolling direction deviated from the light‐guiding path. Also, the spiral ribbon azo‐LCN exhibited intermittent and unstable rolling motion due to the rolling mechanism based on the center‐of‐the‐mass shifting. Conversely, helicoid azo‐LCN undergoes in‐plane twisting deformation due to photo‐induced torsional torque during the winding process, resulting in a helicoidal body with anticlastic curvature. By the torsional torques generated perpendicular to the helix axis, the helicoid shows a continuous and well‐controlled straight rolling motion toward the guiding light. Even with a much smaller rolling diameter, the rolling speed of the helicoid azo‐LCN is comparable to that of the spiral ribbon azo‐LCN due to the considerably higher rotation rate.

Then, a center‐tapered helicoid azo‐LCN locomotor with wide edges was introduced by facilitating the AR‐dependent gradient pitch and axial diameter formation features for faster photo‐motility by overcoming the small effective rolling diameter of the helicoid structure. Moreover, when irradiating the patterned light on the center‐tapered helicoid structure, the rolling direction can be stably and arbitrarily controlled by the macroscopically photo‐guided bending deformation along the helix axis. In addition, the helicoid and center‐tapered helicoid azo‐LCN locomotors allow photo‐steerable multimodal amphibious rolling irrespective of the surface terrain conditions, demonstrated by climbing a steep slope and paddle‐like swimming on a liquid and under a liquid depending on a liquid type. Finally, we demonstrate on‐demand curvilinear photo‐steering of the center‐tapered helicoid azo‐LCN locomotor, which can reach the desired destination by bypassing obstacles via real‐time photo‐guiding control.

## 2 Results and Discussion

### 2.1 Helix‐Structured Azo‐LCN Rolling Soft Locomotors

**Figure 1:**
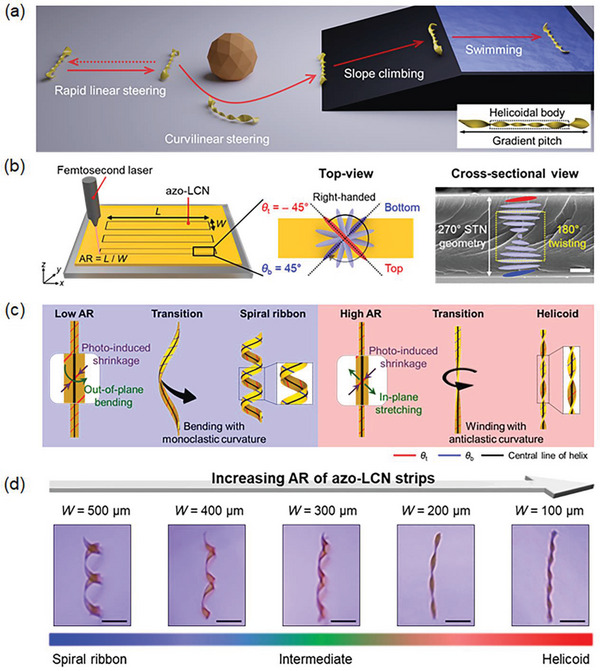
Azobenzene‐functionalized liquid crystal network (azo‐LCN) rolling locomotors with photo‐induced helix structures. a) Schematic of photo‐steerable rolling locomotors controllable on various terrains. b) Schematic illustration and scanning electron microscopy image of azo‐LCN strip with a 270° super‐twisted nematic (STN) geometry. Scale bar: 5 µm. c) Photo‐structured winding process of spiral ribbon and helicoid helix structures according to the aspect ratio (AR) of the azo‐LCN strips. d) Helix structures and their continuous buckling transition according to the AR of the azo‐LCN strips. Sample length: 8 mm, thickness: 20 µm, light intensity: ≈0.2 W cm^−2^ at 365 nm, and scale bar: 2 mm.

In **Figure** **1**a, the photo‐controllable rolling schemes achievable in the center‐tapered helicoid azo‐LCN soft locomotor are shown, depicting rapid forward or backward linear steering enhanced by extended edge‐diameter, curvilinear steering for detouring large obstacles by directional turning, climbing on a steep slope by overcoming gravitational force and slips, and swimming on the liquid by paddle‐like motions. A fast axial rotation is generated in the twisting helicoid azo‐LCN by photo‐fueled torsional torque in the anticlastic‐structured helicoidal body, which is transferred to the wide‐edges self‐structured from a monolithic azo‐LCN strip by AR‐dependent gradient pitch control, thereby exhibiting improved photo‐motility.

For helix‐structured azo‐LCN soft locomotors with rotational photo‐motility, an azo‐LCN film was cut precisely while minimizing edge‐burr issues using a femtosecond laser. The width (*W* = 100–500 µm) was systematically varied, whereas the length (8 mm) and thickness (20 µm) were fixed to regulate the AR of the azo‐LCN soft locomotors in the range from 16 to 80 (Figure 1b). The azo‐LCN film has an anisotropic molecular ordering orientation, achieved by photo‐crosslinked LCNs with an azobenzene derivative molecular switch for photo‐actuation (Figures S1 and S2, Supporting Information). With chiral dopants, the azo‐LCN strips had a 270° STN geometry with right‐handedness, where the molecular orientations at the top and bottom surfaces of the azo‐LCN strips were orthogonally formed at angles of −45° and 45°, respectively (Figure 1b). Upon exposing the chiral azo‐LCN strips to 365 nm UV light, photo‐generated contractive stresses occur orthogonally at the top and bottom surfaces along the nematic director through trans‐cis photo‐isomerization of the azobenzene molecules. In conjunction with directional molecular contraction, macroscopic geometry determines the structures of helices in flat azo‐LCN strips. By introducing 180°‐twisting molecular geometry in the middle layer of the 270° STN structure for rotational stress neutralization, the helix axis was obtained in a more stable straight‐like manner, irrespective of the photo‐deformed rolling curvature type, and was suitable for axial rolling (Figure 1b).

With the same thickness and length, stress competition occurs between the bending energy cost and in‐plane elastic energy cost depending on the width of the chiral azo‐LCN strip, which determines the different global shaping and curvature by photo‐structuring.^[^ ^31^, ^32^, ^33^ ^]^ For lower AR values, the bending energy cost is lower than the in‐plane stretching energy cost in response to photo‐induced anisotropic shrinkage; thus, the out‐of‐plane bending of the azo‐LCN strip induces the winding of the film into a spiral ribbon‐like helix structure with a monoclastic small Gaussian curvature (*K*) (Figure 1c, left; Figure S3a, Supporting Information). Here, the central line of the helix was spirally bent. Conversely, in the case of a higher AR, the in‐plane stretching of the azo‐LCN strip was dominant in the presence of anisotropic photo‐contraction. This causes the generation of torsional torque around the helix axis and forms a helicoid structure that comprises the straight central line of the helix (Figure 1c, right; Figure S3b, Supporting Information). The co‐existence of oppositely signed curvatures provides an anticlastic curvature with a larger Gaussian curvature by avoiding induced stress conflict. By increasing the AR of the monolithic azo‐LCN strip, the helical curvature can be controlled from the spiral ribbon to the helicoid via an intermediate curvature state (Figure 1d). In the anticlastic helicoid structure formed by tight winding around the straight central line of the helix, the average rolling diameter (*D*) and helix pitch (*P*) are quite smaller than those of the global‐shaped monoclastic spiral ribbon (Figure 1d; Figure S4, Supporting Information).

With top‐down UV irradiation on the photo‐structured azo‐LCN helix, photo‐shrinkage and elastic recovery occur periodically along the helix axis on light‐irradiated and light‐shaded surfaces, respectively, with rolling (Figures S5 and S6, Supporting Information). For control along the horizontal direction, a directional UV light source (wavelength *λ* = 365 nm, beam linewidth ≈35 mm) was employed with an angular spreading flux distribution (Figure S7, Supporting Information). Photo‐structuring of the helix and subsequent photo‐mediated rolling actuation was performed above the polymer glass transition temperature (*T_g_*) of *T_g_* + 50 °C where the *T_g_* of the azo‐LCN film was measured to be ≈65 °C by dynamic mechanical analysis (DMA) (Figure S8, Supporting Information). Above *T_g_*, the mechanical stiffness of the azo‐LCN strip can be effectively reduced to facilitate curvature generation and subsequent rolling. In particular, thermal cis‐trans back‐isomerization can be accelerated, regenerating trans isomers for photo‐fueled actuation. Time‐resolved UV transmittance curves (Figure S9, Supporting Information) showed that light transmittance increased more rapidly, thus indicating a 3.5 times higher trans‐cis isomerization rate at *T_g_* + 50 °C compared with the measurement at room temperature (25 °C). In addition, the UV transmittance curve at *T_g_* + 50 °C reached saturation faster than at room temperature, and the UV transmittance level at saturation was lower at the elevated temperature, which is indicative of thermally enhanced cis‐trans back‐isomerization. Hence, the thermal effects induce faster rolling dynamics with accelerated photo‐isomerization at a molecular scale in conjunction with accelerated macroscopic structural turnover frequency for light‐shaded regions. Finally, improved rolling motility is partly supported by introducing a spring‐like 180°‐twisting stress neutralization layer within the 270° STN structure for faster elastic recovery (Figure 1b).

### 2.2 Photo‐Fueled Rolling Motilities: Spiral Ribbon versus Helicoid

**Figure 2:**
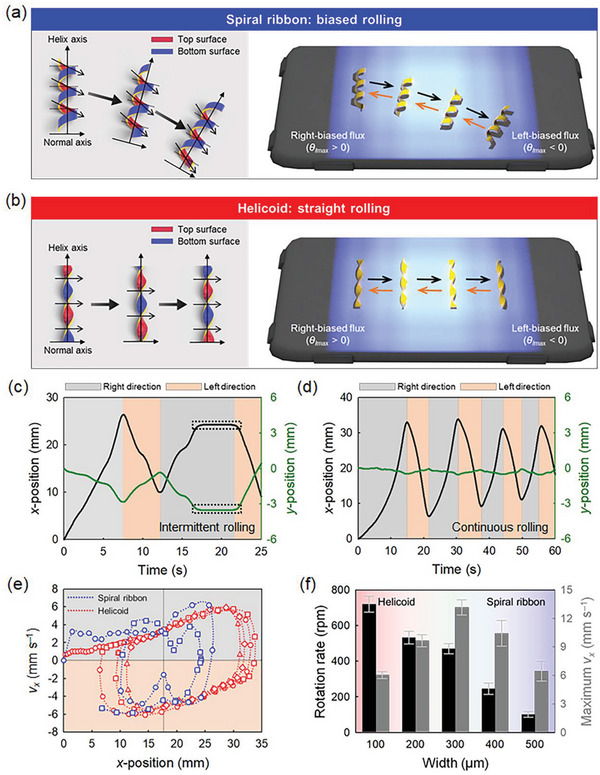
Photo‐fueled rolling characteristics of azo‐LCN helix locomotors. a,b) Schematics of biased and straight rolling mechanisms of spiral ribbon and helicoid helix structures and their round‐trip motion motilities in spatially biased light‐flux conditions. c,d) *x*‐*y* positional changes with time at photo‐actuated repetitive round‐trip motions for c) spiral ribbon and d) helicoid rolling locomotors. e) Trajectories of *x*‐*v_x_* relationships in repetitive round‐trip motions for both chiral structures (○: 1st round trip, □: 2nd round trip, △: 3rd round trip, and ◇: 4th round trip). f) Maximum *v_x_* and rotation rates of rolling locomotors dependent on helix structures obtained by varying the widths of the azo‐LCN strips. Gray and orange backgrounds in the graphs correspond to right‐ and left‐directional rolling motions, respectively. For the horizontal light intensity profile, see Figure S7 (Supporting Information).

Upon directional UV irradiation, we examined the geometric effects of the azo‐LCN helix on the controllability of rolling direction along the short axis of robots and their rolling trajectories during repetitive round trips. Both the spiral ribbon and helicoid move toward the direction of illumination by breaking the symmetry of the elastic body through light irradiation, based on the principle known as the zero‐energy mode (Figures S5 and S6, Supporting Information).^[^ ^5^, ^6^ ^]^ Considering that the rolling movement of both helical structures is toward the light‐irradiated direction, round‐trip photo‐mechanical rolling was achieved by using a lateral gradient light source with respect to the short axis of the azo‐LCN strip. Using a fixed light source, both samples initially shape‐morphed from a flat strip into a helix (spiral ribbon or helicoid), followed by rightward and then leftward rolling for the round‐trip (**Figure** **2**; Figure S10 and Movie S1, Supporting Information). Under the same lateral‐gradient light flux conditions, the rolling directions of the spiral ribbon deviated from the light illumination direction for photo‐guided rolling. Conversely, under identical light conditions, the helicoid azo‐LCN exhibited stable round‐trip rolling motions with considerably improved straightness and directionality concerning the guiding light.

Upon irradiation with obliquely biased light, the top curvature of the monoclastic spiral ribbon became relatively flattened because of photo‐shrinkage effects as the light is exposed to outer surfaces (Figure S5, Supporting Information). Conversely, the bottom curvature is enhanced when inner surfaces are exposed to light. Thus, oblique light results in a macroscopically biased shape deformation and subsequent center‐of‐mass shifting for rolling toward light irradiation direction through a series of repetitions. In a spiral ribbon, the contact line at the substrate is inevitably biased with respect to the helix axis, which induces biased rolling (Figure 2a). For the right‐handed helix, right‐down and left‐up rolling motions were obtained for the round‐trip (Figure S10a and Movie S1, Supporting Information). In addition, the spiral ribbon exhibited unstable intermittent rolling motions because rotational torque was induced by macroscopic structural deformation shifting both the center‐of‐mass and substrate contact line (Figure 2c; Figure S11a, Supporting Information). Thus, the reproducibility of the rolling trajectories decreased when several round trips were conducted (Figure 2e).

Meanwhile, in the anticlastic helicoid azo‐LCN helix structure, the curvature deformation at the exposed regions and the elastic recovery at the unexposed areas generate the collective torsional torque for rolling toward the light irradiation direction without deviation (Figure S6, Supporting Information). In addition, the central line of the helix was not deformed during rolling via the subsequent curvature deformations along the helix axis. The photo‐induced torsional torque of the helicoid can provide a linearly guided rolling motion (Figure S10b and Movie S1, Supporting Information) in the direction of light irradiation. Instead of intermittent rolling, as spiral ribbons exhibit, helicoids demonstrate continuous acceleration and deceleration as the surface contact point shifts along the edge under a fixed light source with a lateral gradient flux distribution (Figure 2d; Figures S11b and S12, Supporting Information). When unbiased light is directed toward a stationary helicoidal structure along the normal direction, the helicoid cannot roll stably due to the symmetric light distribution incident on the helicoidal body and instead folds upward toward the light source (Figure S13, Supporting Information). With highly stable photo‐fueled rolling under biased light irradiation, symmetric trajectories were recorded for repeated round trips (Figure 2e). To reach distant destinations with rolling, the positional intensity profile could be controlled in real‐time by simply moving the light source through the linear motorized stage, wherein the helicoid azo‐LCN helix exhibited directed rolling motility, unlike the spiral ribbon azo‐LCN helix (Figure S14 and Movie S2, Supporting Information).

Rolling motilities were evaluated regarding rotation rate and maximum velocity (*v_x_*) according to the AR of the azo‐LCN strip by systematically varying the width, as summarized in Figure 2f. With increasing AR, the azo‐LCN helices show a structural transition from a spiral ribbon to a helicoid helix, wherein the curvature‐dependent effective rolling diameter monotonically decreases (Figure S4, Supporting Information). Compared with the spiral ribbon (*D* = 1.22 mm at *W* = 500 µm), the tightly wound rolling diameter of the helicoid azo‐LCN is substantially smaller (*D* = 0.16 mm at *W* = 100 µm). However, the *v_x_* of the helicoid azo‐LCN (*v_x_* = 6.03 mm s^−1^) is comparable to that of the spiral ribbon (*v_x_* = 6.43 mm s^−1^), which can be attributed to the much higher rotation rate (≈720 rpm) of the helicoid azo‐LCN. By avoiding the intermittent momentum loss of the spiral, which hinders the acceleration of rolling, the photo‐fueled torsional torque of the helicoid can be transformed efficiently into continuous rotational motion, as represented by the high rotation rate. Under oblique light irradiation, the photo‐structured helicoid helix is momentarily deformed into a biased anticlastic curvature with fast dynamics, deviating from the energetically (+/–) curvature‐competing state,^[^ ^26^ ^]^ and the rotating structure, which is not fixed to a surface, can be quickly re‐stabilized with subsequent rolling motion preserving tightly wound axial stability. This photo‐deforming dynamic process might be an additional reason for the superior photo‐motility achievable in a helicoid rolling locomotor. A helicoid structure with continuous acceleration and directional control without intermittent momentum loss is a more desirable structure for further enhancing photo‐motility speed by improving UV irradiation conditions, such as widening the UV irradiation area or controlling the UV intensity gradient profile. Although the intermediate structure (*W* = 300 µm) between the helicoid and spiral ribbon yielded the highest rolling velocity with a large rolling diameter (0.54 mm) and suppressed intermittent rolling, unstable rolling occurred along the biased direction, which is inappropriate for photo‐guided directional control (Figure S15 and Movie S3, Supporting Information).

### 2.3 Center‐Tapered Helicoid Locomotors: Superior Motility and On‐Demand Steering

**Figure 3:**
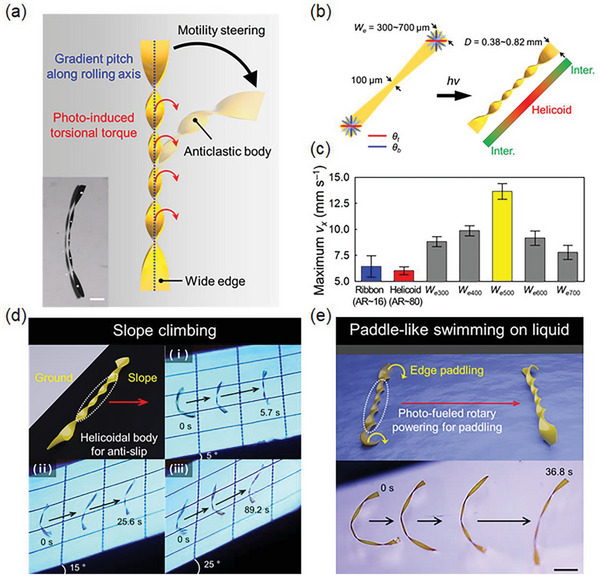
Center‐tapered helicoid locomotor. a) Schematic and snapshot image of center‐tapered helicoid locomotor for agile and steerable photo‐motility. Scale bar: 1 mm b) Initial azo‐LCN strip condition and photo‐structured gradient helix state for a center‐tapered helicoid rolling or swimming locomotor. c) Maximum *v_x_* values according to helix conditions of the spiral ribbon, helicoid, and center‐tapered helicoid structures. d) Climbing on various slopes by a center‐tapered helicoid rolling locomotor (edge width (*W_e_*) = 500 µm, grid length = 5 mm). e) Paddle‐like swimming motion of a center‐tapered helicoid locomotor on the surface of the liquid (glycerol solution, *T* = *T_g_* + 50 °C). Scale bar: 2 mm.

Despite the superior rolling motilities of helicoids, their topology inevitably limits the rolling velocity owing to the smaller helix diameter compared to spiral systems. To circumvent this inherent limitation, we introduce a center‐tapered helicoid structure with wide edges to achieve both a high rotation rate and rolling velocity as well as stable steering capabilities. Notably, the tightly wound helicoid helix generates photo‐fueled torsional torque along the helix axis, similar to the motor‐connected vehicular wheel axle. Based on AR‐dependent helix structuring information (Figure 1d) and the consequent cross‐sectional helix diameter conditions (Figure S4, Supporting Information), the structure is designed to have a wider diameter at both ends while maintaining a helicoidal axis body to enhance rolling velocity (**Figure** **3a**). By simply introducing a width‐gradient azo‐LCN strip, center‐tapered helicoid locomotors can be constructed from a single monolithic film. The photo‐motilities of center‐tapered helicoid locomotors were investigated by precisely and systematically varying the edge width (*W_e_*) from 300 to 700 µm at a fixed central width of 100 µm via laser cutting (Figure 3b; Figure S2, Supporting Information). Because of the width‐dependent helical curvature formation via stress competition, a helicoidal anticlastic helix was formed for the rotational‐torque‐generating like wheel axle, while larger edge diameters were formed in conjunction with a gradient pitch along the helix axis from the axis center to the edges (Figure S16, Supporting Information). Compared with the *v_x_* of the helicoid rolling soft locomotor, the center‐tapered helicoid locomotors exhibited a higher *v_x_*, originating from a larger helix edge diameter (Figure 3c). By increasing *W_e_* up to 500 µm, the rolling velocity reached 13.63 mm s^−1^, 2.3 times higher than that of a helicoidal locomotor with a uniform helix diameter (*W* = 100 µm). Interestingly, even with a smaller helix diameter (*D* = 0.63 mm from *W_e_* = 500 µm) than that of the spiral ribbon (*D* = 1.22 mm from *W* = 500 µm), the center‐tapered helicoid locomotors can exhibit doubled rolling motility because photo‐fueled rolling was generated by the photo‐efficient rotational torque at the tightly wound helicoidal axle. The rotation rate of the center‐tapered helicoid locomotors (*W_e_* = 500 µm) was ≈413 rpm, which was lower than that of the regular helicoid (≈720 rpm at *W* = 100 µm). This can be attributed to the rolling hindrances at the edges where the cross‐sectional edge shapes deviate from the ideal circular shape in curved shaping as *W_e_* increase. For the same reason, *v_x_* decreases with increasing edge diameter for *W_e_* > 500 µm (Figure 3c). By increasing *W_e_* at a fixed center width and strip length, the portion of the torque‐generating helicoid decreases in the rolling axle. This can also be a reason for the decrease in *v_x_* for *W_e_* > 500 µm. Linear and stable guided rolling was also obtained for *W_e_* up to 500 µm with the tightly wound helicoid of the wheel axle (Figure S17, Supporting Information).

The intensity of irradiated UV light and the temperature level of the substrate affect the trans‐cis isomerization rate and cis‐trans back‐isomerization rate of photo‐responsive azobenzene molecule, and the rolling velocity of the azo‐LCN locomotor changes according to those conditions. The maximum intensity value in the center position of the beam spot area was controlled while maintaining the intensity profile shape, and the *v_x_* of the helicoid (*W* = 100 µm) and center‐tapered helicoid (*W_e_* = 500 µm) structures were evaluated. For both photo‐actuated helix structures, the *v_x_* increases as the investigated UV intensity conditions increase due to the enhanced effects of the photo‐isomerization recycling rate. As the UV intensity increases from 0.06 to 0.22 W cm^−2^, the *v_x_* of the helicoid helix rolling increases from 0.93 to 6.03 mm s^−1^, and it could be improved from 3.18 to 13.63 mm s^−1^ in the case of the center‐tapered helicoid helix rolling (Figure S18, Supporting Information). Furthermore, we also evaluated the photo‐induced rolling motility by varying substrate temperature conditions. In both helix structures, the maximum rolling velocities increase with increasing the substrate temperature condition within the temperature range (75–115 °C) applied in our experiment (Figure S19, Supporting Information). Young's modulus of the azo‐LCN film decreases as the substrate temperature increases, which provides favorable conditions for the photo‐induced formation of the anticlastic curvature morphological transformation required for photo‐actuated rolling motility in both helicoid and center‐tapered helicoid helix structures (Figure S8, Supporting Information). Additionally, as explained in Figure S9 (Supporting Information), increasing the temperature conditions of the azo‐LCN film increases the photo‐isomerization recycling dynamics, allowing for faster‐rolling motility at a higher substrate temperature. For the helicoid helix, the *v_x_* increased from 0.71 to 6.03 mm s^−1^ as the temperature increased from 75 to 115 °C, while the center‐tapered helicoid helix structure, it increased from 2.71 to 13.63 mm s^−1^. For all the experiment's UV intensities and temperature conditions, the *v_x_* levels were significantly higher in the center‐tapered helicoid helix structure, designed to have a larger effective rolling diameter along the helix axis, than in the helicoid helix structure.

A climbing modality on a slope with rolling requires a strong rotational force to overcome the gravitational force upon a moving body, in conjunction with anti‐slip properties in its direct interaction with the inclined surface. In the case of the spiral ribbon adopted in the previous approach,^[^ ^28^ ^]^ climbing modalities are weakened considerably because both the center of mass shifting and asymmetric curvature at the substrate interface on a slope depend highly on the slope angle. However, the presented helicoid and center‐tapered helicoid locomotors could climb a slope with a rolling modality at much faster axial rotations in the anticlastic helicoid case. Thus, a steeper slope (up to 25° in our evaluations) could be climbed with a superior climbing modality by the helicoid and center‐tapered helicoid locomotors (Figure 3d; Figure S20 and Movie S4, Supporting Information). Here, the screw‐shaped anticlastic body of the rolling axis may play an additional critical role in preventing slip through the interaction between the distributed screw‐shaped bottom edges and the slope surface. Similar to its rolling properties on a flat surface, a center‐tapered helicoid climbed faster than a helicoid locomotor (Figure S21, Supporting Information). By increasing the inclination angle, the average climbing velocities decreased because of the increased effective weight force, and the enhancement effect of the climbing motilities decreased because of the increased body mass of the center‐tapered helicoid structure (≈0.12 mg) compared to the helicoid case (≈0.04 mg) (Figure S21, Supporting Information). In addition, the climbing direction can be photo‐guided for helicoid and center‐tapered helicoid structures. However, a locomotor with an intermediate helix structure (*W* = 300 µm) (Figure 1d) generates a biased rolling motion that deviates from the slope direction, which is undesirable for overcoming slope obstacles. The photo‐activated global curvature deformation is unsuitable for stable surface contact of a climbing body that is expected to have anti‐slip capabilities (Figure S22, Supporting Information).

The photo‐fueled rotational torque generated along the helix axis of the helicoid can be effectively utilized for amphibious rolling or swimming motions in both rigid (flat or inclined surfaces) and liquid. The spiral ribbon failed to swim in liquid, wherein the liquid environment was easily deformed at the interface of the soft locomotor in response to the photo‐activated center‐of‐mass shifting and global curvature shaping. This may be one of the reasons why swimming motion in liquid for amphibious modalities has rarely been reported. Conversely, photo‐fueled axial rotation of the helicoid or center‐tapered helicoid locomotors can be effectively transduced into propulsion‐driven swimming in liquid (Figure 3e) for amphibious modalities of a monolithic film. Considering the operating temperature (*T_g_* + 50 °C), we used glycerol solution (5.87 cP at *T_g_* + 50 °C) and silicone oil solution (26.12 cP at *T_g_* + 50 °C) with different viscosity as liquid media. Compared with the rigid surface case, the photo‐structured helicoidal pitch became looser, and photo‐fueled swimming velocity thus decreased in both solutions (Figure 3c–e; Figure S23 and Movie S5, Supporting Information). The curved edges acted as rotating paddles to support swimming motility (Figure 3e). However, the swimming behaviors differed significantly depending on the liquid conditions. In the glycerol solution condition, paddle‐like swimming was obtained for the helicoid helix and center‐tapered helicoid helix azo‐LCN locomotors, maintaining a floating state on the liquid surface. In contrast, in the silicone oil condition, the paddle‐like motion caused the sample to gradually penetrate into the silicone oil as the paddle‐like swimming progressed, eventually swimming under the liquid. These two distinctly different swimming behaviors can be attributed to the difference in buoyancy effects due to the relatively low density of the silicone oil (1.05 g mL^−1^) compared to the glycerol solution (1.25 g mL^−1^). However, more importantly, the different surface wettability characteristics between the azo‐LCN helix surface and two liquids can be considered as the reason (Figure S24, Supporting Information). When comparing the swimming motility characteristics under photo‐actuation in the two liquid conditions, it can be observed that swimming under the relatively high viscosity condition of the silicone oil results in a much faster swimming velocity (Figure S23 and Movie S5, Supporting Information). Unlike the rolling motion on a rigid surface, in fluid conditions, motion is formed by the physical interaction between the helix and the surrounding fluid. Thus, under the viscosity range conditions of the two liquids employed in the experiment, the higher viscosity of silicone oil provides a more favorable condition for obtaining faster swimming motility characteristics. Furthermore, swimming by the paddle‐like helix rotation under the liquid, rather than swimming on the surface, allows for a wider effective surface area in contact with the surrounding fluid, making it more effective for movement.

**Figure 4:**
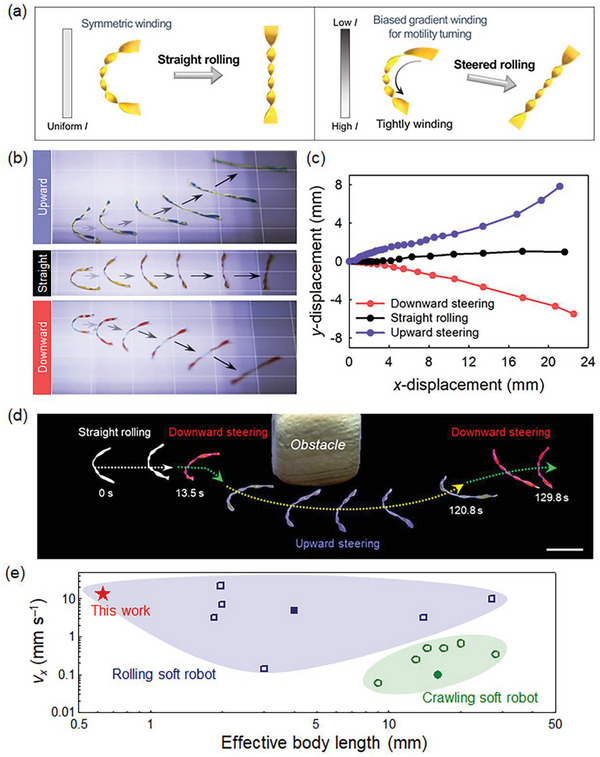
On‐demand steering of center‐tapered helicoid rolling locomotor. a) Symmetric and asymmetric initial winding of helicoidal helix axis according to light intensity profile for on‐demand rolling directional steering. b) Snapshot images of photo‐steered upward or downward curvilinear and straight rolling motions (grid length = 5 mm). c) *x*‐*y* displacement graphs for each photo‐steering condition. d) On‐demand photo‐guided agile rolling motions to reach the destination by detouring a large obstacle via real‐time light source control. Scale bar: 5 mm. e) Comparison of photo‐motility by plotting *v_x_* with respect to moving‐related effective body length for various types of photo‐actuated rolling^[^ ^11^, ^28^, ^29^, ^30^, ^34^, ^35^, ^36^ ^]^ or crawling^[^ ^22^, ^23^, ^24^, ^37^, ^38^, ^39^, ^40^ ^]^ soft robots (filled and unfilled symbols: with and without photo‐steerable function, respectively).

Helicoidal locomotors can provide photo‐guided direction‐steerable rolling by selecting the rolling directions and detouring steep and large obstacles that cannot be overcome with climbing. When the light was irradiated obliquely concerning the moving path with a uniform intensity profile along the helix‐axis direction, symmetric bending initially appeared in the center‐tapered helicoid locomotor owing to the width‐dependent gradient pitch condition from the center to the edges (Figure 1d). The helicoid became tighter, and the helix axis straightened, resulting in faster photo‐guided straight rolling motions (**Figure** **4**a). Conversely, with a gradient intensity profile, the photo‐structured helix axis initially undergoes asymmetric bending owing to the biased gradient helix pitch. The latter had a tighter winding in the higher‐intensity region (Figure S25, Supporting Information). This induces steering effects, and the center‐tapered helicoid locomotor attains fast and linear rolling motions after tight winding is achieved in the photo‐steered direction. When we irradiate a gradient light on the helicoid structure without widened edges, the photo‐structured helicoid is severely folded along the helical axis near the high‐intensity region instead of undergoing asymmetric bending for the steering motion, which is the result of biased axial photo‐deformation escaping from the energy‐balanced condition of the anticlastic helicoid helix. In contrast, wide edges can prevent severe axial folding, which provides stable asymmetric axial bending for the photo‐steered rolling modality. Note that the spiral ribbon formed as monoclastic small Gaussian curvatures with out‐of‐plane bending is unsuitable for providing the helical axis bending required for rolling‐direction steering (Figure 1c, left). By switching the intensity profiles or the bias direction of the gradient intensity profile, horizontal linear rolling and upward or downward steered rolling motions were selectively conducted (Figure 4b,c; Movie S6, Supporting Information). By moving the actinic light with in situ monitoring of the rolling sample position, clockwise and counter‐clockwise circular movements can be selected (Figure S26, Supporting Information). Finally, a real‐time on‐demand photo‐steerable rolling locomotor is presented with a center‐tapered helicoid controlled by moving the actinic light (Figure 4d; Movie S7, Supporting Information) through the selective in situ combination of straight linear rolling for agile movement and curvy‐steered rolling for detouring large obstacles to reach a destination quickly and successfully.

The proposed center‐tapered helicoid locomotor exhibited excellent rolling motility at extremely low body mass (≈0.12 mg) and very small rolling diameters (≈0.63 mm) compared with previous photo‐actuated soft robots (Figure 4e).^[^ ^11^, ^22^, ^23^, ^24^, ^28^, ^29^, ^30^, ^34^, ^35^, ^36^, ^37^, ^38^, ^39^, ^40^ ^]^ Moreover, it offered multifunctionality driven by light, enabling scalability in diverse driving environments like inclined surfaces and liquids, as well as on‐demand real‐time photo‐steering (Table S1, Supporting Information).

## 3 Conclusion

Agile and steerable photo‐fueled rolling locomotors driven by helicoidal torsional torque were demonstrated using a curvature engineering process implementable with monolithic azo‐LCN films. Photo‐actuated azobenzene molecules were self‐assembled with LC directors into a 270° STN molecular chiral geometry for photo‐induced helix structures, and improved photo‐fueled rolling dynamics were achieved. Using the same chiral molecular geometry along the depth direction of the azo‐LCN film, various photo‐induced helical structures from the spiral ribbon (at lower AR values) to the helicoid (at higher AR values) were constructed depending on the AR. Spiral ribbon structures were formed by out‐of‐plane bending along the strip, resulting in larger diameters advantageous for rolling. However, intermittent and biased rolling motions, deviating from photo‐guidance, occur due to rolling mechanisms associated with macroscopic curvature deformations. Conversely, helicoid structures, formed by in‐plane twisting of the strips, effectively generated photo‐fueled torsional torque during the axial winding process, as evident from the much higher rotational velocity (≈720 rpm at *W* = 100 µm) and high rolling velocity (*v_x_* = 6.03 mm s^−1^) even with a much smaller helix diameter (*D* = 0.16 mm) for rolling compared with that of the spiral ribbon (*D* = 1.22 mm at *W* = 500 µm). In addition, helicoid locomotors can generate continuous linear rolling motions following photo‐guided directions.

Based on the curvature engineering of monolithic azo‐LCN strips, photo‐motility of the helicoid structure can be enhanced considerably by introducing center‐tapered structures with a larger axial diameter at the edges. The center‐tapered helicoid locomotor (*W_e_* = 500 µm), wherein the rotation was mainly controlled by the helicoid helix axis directly connected to the edges, attained a doubled rolling velocity (13.6 mm s^−1^) than that of the helicoid structure (*W* = 100 µm) made without the center‐tapered AR design scheme while the center‐tapered helicoid locomotor maintains continuity in photo‐guided rolling motility. The center‐tapered helicoid locomotors can provide multimodal amphibious motilities at various surface conditions. They can climb a steep slope by overcoming the weight force and preventing slipping. In swimming photo‐modality, the curved edge can act as photo‐fueled rotating paddles, enhancing swimming motility. The center‐tapered helicoid structure also provides better geometrical conditions for direction control of photo‐steered rolling via asymmetric axial bending. Programmable photo‐mechanical steering behavior has been successfully demonstrated with real‐time on‐demand guiding light control to circumvent obstacles by conducting selective rolling modality controls of straight and curved steering. This unprecedented helix‐curvature engineering strategy will provide insights into the unconstrained rolling maneuverability and capabilities of miniaturized soft robots operated by untethered remote control.

## 4 Experimental Section

### Synthesis and Fabrication

A photo‐responsive molecular machine, 4,4′‐bis(6‐(acryoloxy)hexyloxy)azobenzene (Synthon Chemical), was mechanically mixed with the reactive liquid crystal monomer, 4‐(3‐acryloyloxypropyloxy)‐benzoic acid 2‐methyl‐1,4‐phenylene ester (RM257, Merck), photoinitiator (Irgacure 784, Ciba), and a chiral dopant (R811, Merck) at 20, 78.8, 1, and 0.2 wt.%, respectively (Figure S1, Supporting Information). The mixture was heated to 120 °C in an isotropic state and inserted into a cell (thickness = 20 µm) in which the top and bottom substrates had alignment axes equal to –45° and 45°, respectively (Figure 1b; Figure S2, Supporting Information). The cell temperature was maintained at 75 °C for the nematic state of the molten mixture, and the molten mixture in the glass cell was photo‐polymerized by a light‐emitting diode (LED) light (*λ* = 532 nm, intensity of ≈0.05 W cm^−2^, 1 h). Azo‐LCN films were harvested from the glass cell and cut into strips of various aspect ratios and shapes using a femtosecond laser (TruMicro 2030, Trumpf).

### Temperature‐Dependent Time‐Resolved UV Transmittance

To characterize and compare the temperature‐dependent trans‐cis photo‐isomerization and cis‐trans back‐isomerization dynamics properties of the azo‐LCN films, time‐resolved UV transmittance curves were measured at room temperature (25 °C) and elevated temperature (*T_g_* + 50 °C = 115 °C). For this characterization, azo‐LCN cells (thickness = 4 µm) were prepared under antiparallel alignment conditions on the top and bottom alignment surfaces. Shape deformation in actinic light irradiation was suppressed by preparing azo‐LCN films between two rigid glass substrates. The input light source for trans‐cis photo‐isomerization activation was a collimated LED (SST‐10‐UV, Luminus) (*λ* = 365 nm, intensity of ≈0.05 W cm^−2^). To measure the temperature‐dependent time‐resolved UV transmittance, azo‐LCN cells were set within a high‐precision temperature controller (HCS302/STC200, Instec Inc.) with a transparent window (Figure S9a, Supporting Information). The time‐dependent transmitted light intensity was measured under both temperature conditions using a photo detector (Model 2031, Newport Inc.) and an oscilloscope (DSO1052B, Keysight Technologies Inc.).

### Photo‐Mechanical Rolling and On‐Demand Steering

Custom‐designed UV LED arrays (*λ* = 365 nm, SST‐10‐UV, Luminus) with a narrow spectral bandwidth (full‐width‐half‐maximum Δ*λ* ∼10 nm) were used for photo‐structuring and photo‐mechanical rolling actuation of the azo‐LCN soft locomotors. For directed and repetitive round‐trip rolling motions, UV LED arrays were designed to have horizontal gradient intensity profiles for light‐guided rolling using angular‐dependent directional UV irradiation (Figure S7, Supporting Information). Azo‐LCN strips and azo‐LCN soft locomotors were placed on a heating plate at a surface temperature of *T_g_* + 50 °C. In the experiment analyzing the rolling velocity according to surface temperature (Figure S19, Supporting Information), the *T_g_* + 10 °C to *T_g_* + 50 °C was used. The directional UV light source was equipped with a linearly motorized stage (LTS 150/M, Thorlabs) to achieve angular UV flux control through the *x*‐*y* translation of the UV source with respect to the sample position. For unidirectional or round‐trip rolling, the UV source was set to a fixed biased position concerning the initial sample‐loading position (Figure S7, Supporting Information). For on‐demand steered rolling, the UV source position was controlled in situ using the biaxial operation of the *x*‐*y* translation stage during real‐time monitoring of the rolling samples. The photomechanical rolling motion of the azo‐LCN soft locomotor was captured using a high‐precision DSLR camera (EOS80D, Canon).

### Climbing on Slope and Swimming in Liquid

For slope‐climbing of the rolling soft locomotors, a wooden board was set at various inclination angles from 5° to 25° (Figure 3d; Figure S20, Supporting Information). To provide the same surface temperature conditions as the rolling experiments using a flat surface, the slope surface was heated to *T_g_* + 50 °C. For swimming motion studies, glycerol (Sigma–Aldrich) and silicone oil (175633, Sigma–Aldrich) solutions were used, and the temperature of solutions was controlled to be *T_g_* + 50 °C. The viscosity of each solution at the temperature of *T_g_* + 50 °C was measured using a rheometer (ARES‐g2, TA instrument). The glycerol and silicone oil solutions did not react chemically with the azo‐LCN locomotors.

## Conflict of Interest

The authors declare no conflict of interest.

## Supporting information

Supporting Information

Supplemental Movie 1

Supplemental Movie 2

Supplemental Movie 3

Supplemental Movie 4

Supplemental Movie 5

Supplemental Movie 6

Supplemental Movie 7

## Data Availability

The data that support the findings of this study are available from the corresponding author upon reasonable request.
