# Supplementary material for: Steerable and Agile Light‐Fueled Rolling Locomotors by Curvature‐Engineered Torsional Torque
Source: Adv Sci (Weinh). 2023 Aug 11;10(30):2304715. doi: 10.1002/advs.202304715 (PMC10602523; doi:10.1002/advs.202304715)
Supplement: Supplementary file 1 — Supporting Information [file ADVS-10-2304715-s006.pdf]

ADVANCED SCIENCE
Open Access

Supporting Information

for *Adv. Sci.*, DOI 10.1002/advs.202304715

Steerable and Agile Light-Fueled Rolling Locomotors by Curvature-Engineered Torsional Torque

Jun-Chan Choi, Jisoo Jeon, Jae-Won Lee, Asad Nauman, Jae Gyeong Lee, Woongbi Cho, Chanwoo Lee, Young-Min Cho, Jeong Jae Wie and Hak-Rin Kim**

Supporting Information

Steerable and Agile Light-Fueled Rolling Locomotors by Curvature-Engineered Torsional Torque

Jun-Chan Choi[†]*, Jisoo Jeon*[†]*, Jae-Won Lee, Asad Nauman, Jae Gyeong Lee, Woongbi Cho, Chanwoo Lee, Young-Min Cho, Jeong Jae Wie*[*]*, and Hak-Rin Kim*[*]

[†]These authors contributed equally to this work.

J.-C. Choi, J.-W. Lee, A. Nauman, C. Lee, Prof. H.-R. Kim
School of Electronic and Electrical Engineering
Kyungpook National University
Daegu 41566, Republic of Korea
E-mail: rineey@knu.ac.kr (H.-R. Kim)

J.-C. Choi
Soft Hybrid Materials Research Center
Korea Institute of Science and Technology
Seoul 02792, Republic of Korea

J. Jeon
Program in Environmental and Polymer Engineering
Inha University
Incheon 22212, Republic of Korea

J. G. Lee, W. Cho, Prof. J. J. Wie
Department of Organic and Nano Engineering
Hanyang University
Seoul 04763, Republic of Korea
E-mail: jjwie@hanyang.ac.kr (J. J. Wie)

J. G. Lee, W. Cho, Prof. J. J. Wie
Human-Tech Convergence Program

Hanyang University
Seoul 04763, Republic of Korea

Y.-M. Cho, Prof. H.-R. Kim
School of Electronics Engineering
Kyungpook National University
Daegu 41566, Republic of Korea

Prof. J. J. Wie
Department of Chemical Engineering
Hanyang University
Seoul 04763, Republic of Korea

Prof. J. J. Wie
Institute of Nano Science and Technology
Hanyang University
Seoul 04763, Republic of Korea

Prof. J. J. Wie
The Research Institute of Industrial Science
Hanyang University
Seoul 04763, Republic of Korea

Prof. J. J. Wie
The Michael M. Szwarc Polymer Research Institute
State University of New York College of Environmental Science and Forestry
Syracuse, NY 13210, USA

Prof. J. J. Wie
Department of Chemical Engineering
State University of New York College of Enviromental Science and Forestry
Syracuse, NY 13210, USA

Photo-Structured Helix-Morphing of Azo-LCN Strip

Helix structures formed by azobenzene-functionalized liquid crystal polymer network (azo-LCN) strips are caused by photo-induced anisotropic mechanical stresses through the photo-isomerization process of azobenzene molecules and are determined by the stress-generating principal axis distributions, those are parallel to the photo-responsive azo-LCN alignment directions and their relative angles (φ) with respect to the geometric axes of the azo-LCN strip (Figure 1c). In this study, the azo-LCN strip has molecular alignment axes perpendicular to each other at the top and bottom surfaces and has a 270° super-twisted nematic (STN) geometry with right-handedness along the film thickness direction by doping chiral dopants (Figure 1b; Figure S1, Supporting Information). As discussed in Figure 1, by varying the aspect ratio (AR) condition of the azo-LCN strip, the helix geometry can be selectively chosen, meaning that the helix-based rolling locomotor structure can be changed from a spiral ribbon to a helicoid structure from the monolithic azo-LCN strip. This can be achieved by changing the width (W) condition of the strip while keeping the same strip length (8 mm) and thickness (20 µm) conditions. To obtain enhanced photo-guided directionality and photo-activated rolling motility, it is desirable to have a helix axis of the photo-structured helix geometry, formed by UV exposure, in a straight-line form rather than a bending one. To achieve this, it is necessary to set the relative angle condition of φ between the azo-LCN alignment directions, determining the photo-induced stress principal axes, and the major axis of the strip appropriately during the design and fabrication process of the azo-LCN strip (Figure S2, Supporting Information).

In the Euler curvature formula for analyzing helix geometries, the normal curvature is defined as $\kappa_n = \kappa_1\cos^2\varphi + \kappa_2\sin^2\varphi$, where κ_1 and κ_2 are two principal curvatures.[33] In the spiral ribbon helix obtainable at a small AR condition, κ_n is the positive constant (Figure 1c; Figure S3a, Supporting Information), and φ needs to be $\varphi \neq 0$. At φ is set to 0, the photo-structured curvature shape tends to form a ring shape rather than a stretched helix structure of spiral ribbons.[28] The helicoid helix, obtainable at a high AR condition, has two non-zero principal curvatures of κ_1 and κ_2 with opposite signs, generating anticlastic curvature deformation (Figure 1c; Figure S3b, Supporting Information). Following the Euler curvature formula, for an ideal anticlastic helicoid helix forming the straight-line form of both the central line of the helix and the helix axis, κ_n needs to be zero, which is obtained when both principal curvatures have equal absolute value conditions ($\kappa_2 = -\kappa_1$) and φ becomes $\pm\pi/4$ of the relative angle conditions.[31,33] Thus, compared with the photo-structured spiral ribbon helix, the precise control of φ is necessary in achieving improved properties of both photo-guided directionality

and photo-activated rolling motility with the photo-structured helicoid helix. In our experiment, as shown in Figure S2, Supporting Information, the φ conditions are precisely determined by the laser-cut process and the molecular alignment direction control of the azo-LCN strip. When the φ condition deviates from the $\pm\pi/4$ condition, it becomes difficult to obtain an ideal helicoid helix with a straight-line form of the central line of the helix, which is required for a tightly twisted helicoid helix. This leads to a decrease in photo-motility characteristics. In addition, the helix axis appears as a bending structure rather than a straight-line form, which, along with the decreased photo-motility characteristics, causes structural instability in the photo-structured formation of an anticlastic helix structure.

At the condition of $\varphi = \pm\pi/4$, the helix structure, from spiral ribbon to helicoid, is determined by the competition between bending energy and stretching energy induced by photo-induced shrinkage along the molecular alignment axis affected by the width of the azo-LCN strip.[31,33] For azo-LCN strips with a large *W*, bending deformation has a lower energy cost compared to a stretching one, leading to a spiral ribbon helix structure through out-of-plane bending deformation forming monoclastic small Gaussian curvature ($K = \kappa_1\kappa_2$) close to 0 (Figure 1c; Figure S3a, Supporting Information). As the *W* decreases, stretching deformation becomes less energy cost compared to bending one, resulting in a twisting deformation due to in-plane stretching (Figure 1c). This deformation leads to the formation of a helicoid helix structure with a straight form of the center line of the helix and anticlastic negative ($K < 0$) Gaussian curvature (Figure S3b, Supporting Information).

Photo-Actuated Rolling Mechanisms of Azo-LCN Locomotor

As shown in Figure S5, Supporting Information, without considering the biased light irradiation effects, the cross-sectional shape of the photo-structured spiral ribbon would be a symmetric circle. However, when photo-activating light is irradiated in the right-biased direction, the convex curvature, light-illuminated on the outer surface, becomes flattened, while the concave curvature is enhanced. As a result, the center of mass of the spiral ribbon cross-section shifts toward the light irradiation direction, and the tangent vector at the surface contact interface also becomes asymmetric. As the angle-biased light irradiation continues, the rotational torque induced by symmetry breaking gradually increases, and when it reaches a certain threshold level, the rolling motion of the spiral ribbon occurs. The threshold torque level is also determined by the interfacial asymmetry of the contact surface at the substrate as well as by the degree of the center-of-mass shift. Because two different rolling mechanisms are involved with different photo-dynamics in the photo-induced rolling of the spiral ribbon helix, the time interval for reaching the rolling threshold condition cannot be identical and

continuous, and intermittent rolling motion easily occurs (Figure S11a, Supporting Information). Importantly, the photo-guided rolling direction is inevitably biased by the inclined surface contact line between the substrate and the spiral ribbon (Figure 2a, c).

Unlike spiral ribbon structures, the rolling motion of the helicoid helix structure is driven by photo-induced torsional torque accompanied by local curvature changes in the lighting and shading areas. Without considering the biased light illumination condition, in an ideal helicoid structure, it can be assumed that the two principal curvature components, κ_1 (>0) and κ_2 (<0), which form the anticlastic curvature, have opposite signs but identical absolute values with similar curvature deformation amounts. To analyze the photo-guided rolling properties of the helicoid helix structure, the anticlastic body within a helicoid helix node is divided into four quadrants, and the photo-induced curvature deformation of each quadrant is defined as κ_a, κ_b, κ_c, and κ_d (Figure S6, Supporting Information). Depending on the bias direction of the light irradiation, the lighting and shading areas of the helicoid body are determined. With the right-biased light irradiation, in the lighting areas on the helicoid surfaces, the absolute value of negative curvature $\kappa_{2,a}$ increases, and that of positive curvature $\kappa_{1,b}$ decreases due to the light-induced surface contraction through the trans-cis photo-isomerization process of the azobenzene molecules, while in the shading area observed at the top view, each curvature is adjusted by the curvature change of the adjacent lighting region as well as the stress recovery through the cis-trans back-isomerization, leading to the decrease of the absolute value of the negative curvature $\kappa_{2,d}$ and the increase of the absolute value of the positive curvature $\kappa_{1,c}$. When examining the curvature-enhancing and curvature-suppressing regions within the helicoid helix under the angle-biased light irradiation conditions, as presented in Figure S6, it can be observed that the curvature asymmetries occur along both principal curvature axes (κ_1 and κ_2) with respect to the light irradiation direction. At the right-biased light irradiation, the helicoid surfaces on the left side of the helix axis line exhibit curvature enhancement, while those on the right side of the helix axis line exhibit curvature suppression. Furthermore, when the direction of light irradiation is reversed, meaning the left-biased light irradiation, the curvature enhancement and suppression positions are reversed with respect to the helix axis. This means that, by switching the direction of the biased light irradiation to the right or left, helicoid helix rotation in the clockwise or counterclockwise direction can be selected, implying that the rolling direction can be controlled by manipulating the light irradiation direction.

Figure S12, Supporting Information, shows helicoid helix structures during photo-induced rolling. Periodic contact points with the substrate surface continuously shift along the

helicoid helix's cross-sectional edge line. At any moment during the rolling process, when viewed from the same selected region along the helix axis, the cross-section sets of the helix rotate clockwise in both the light irradiation region and the light shadow region by the curvature deformation.

The experimental light irradiation condition with a biased angle, for photo-structuring helicoid helix or center-tapered helicoid helix and obtaining their photo-activated rolling motilities, is not only necessary for controlling the rolling direction but also has a significant impact on stabilizing the helicoid helix formation and improving the photo-motility characteristics. Figure S13, Supporting Information, shows positional curvature characteristics and their changes in the helicoid helix structure with the UV radiation condition, normally on the substrate and sample. When the light is normally irradiated, the absolute values of positive curvatures both of $\kappa_{1,\mathrm{b}}$ and $\kappa_{1,\mathrm{c}}$ decrease with suppressing curvature along the κ_1 curvature line. Whereas the absolute values of negative curvatures both of $\kappa_{2,\mathrm{a}}$ and $\kappa_{2,\mathrm{d}}$ increase with enhancing curvature along the κ_2 curvature line. This results in a positional curvature deformation pattern that is difficult to achieve photo-induced rolling biased in a specific direction. Although photo-induced helix structuring was occasionally observable under experimental conditions where perfect sample symmetry was difficult to achieve, it was difficult to obtain the photo-induced rolling characteristic stably. Under this normal irradiation condition, macroscopic bending along the helix axis or severe axial deformation of upward folding normal to the substrate surface, as shown in Figure S13, Supporting Information, was often observed rather than photo-induced rolling. This can be understood by considering the curvature suppressing along the κ_1 curvature axis together with the curvature enhancing along the κ_2 curvature axis, leading to an abrupt curvature transition from the anticlastic to monoclastic curvature with folding upward at random positions within the helix due to the accumulated photo-induced stress.

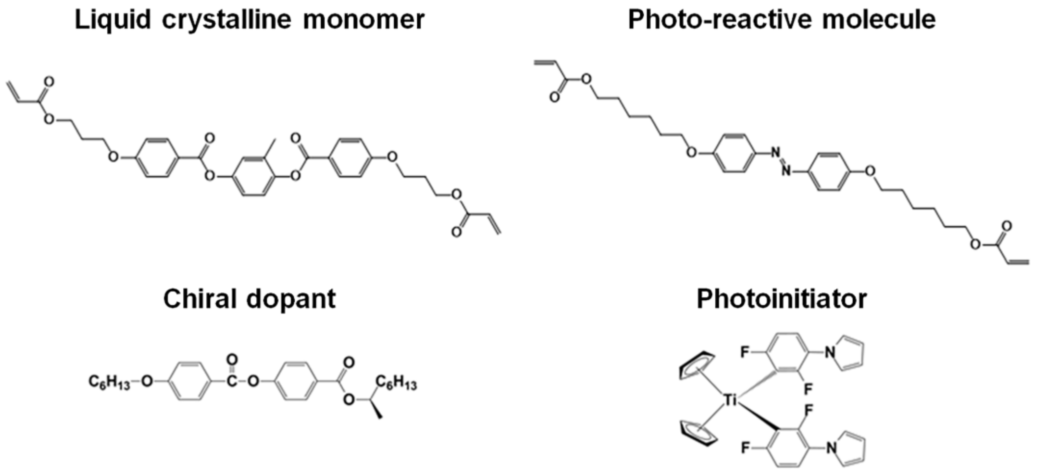

Figure S1. Chemical components of an azobenzene-functionalized liquid crystal polymer network (azo-LCN) film.

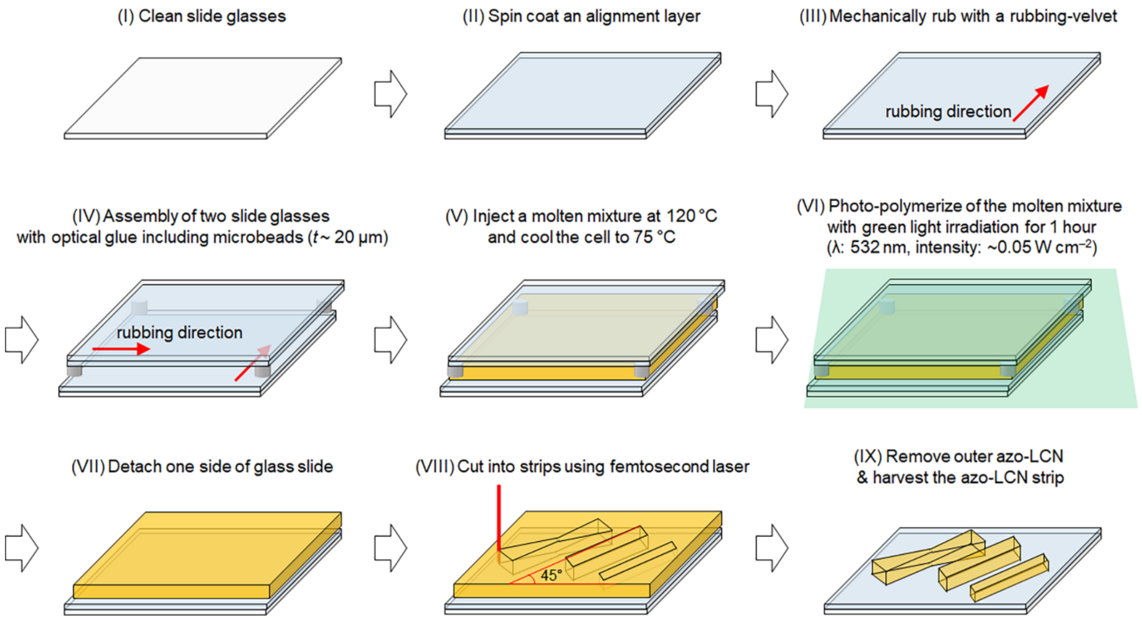

Figure S2. Schematic illustration of azo-LCN strips preparation for photo-actuated rolling locomotors.

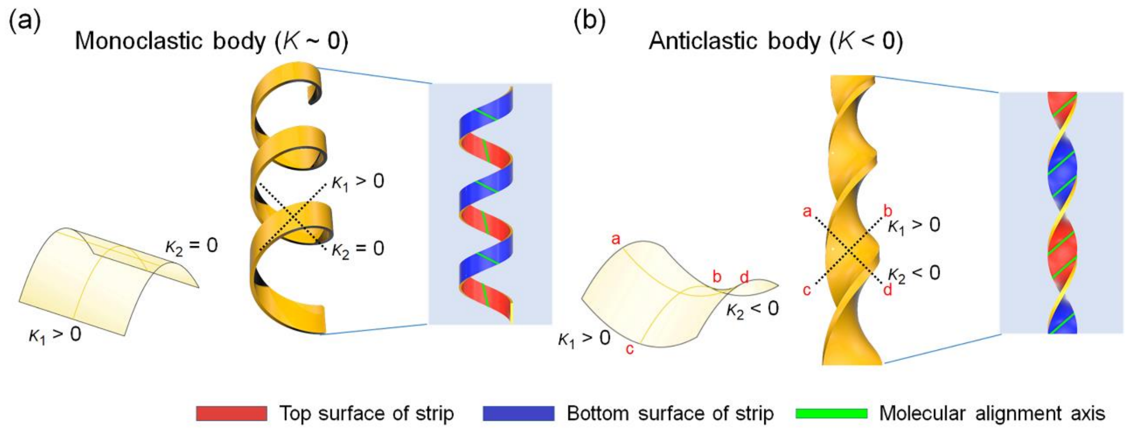

Figure S3. Photo-structured azo-LCN helix structures of (a) spiral ribbon with monoclastic curvature ($\kappa_1 \cdot \kappa_2$ ~0) and (b) helicoid with anticlastic curvature ($\kappa_1 \cdot \kappa_2 < 0$).

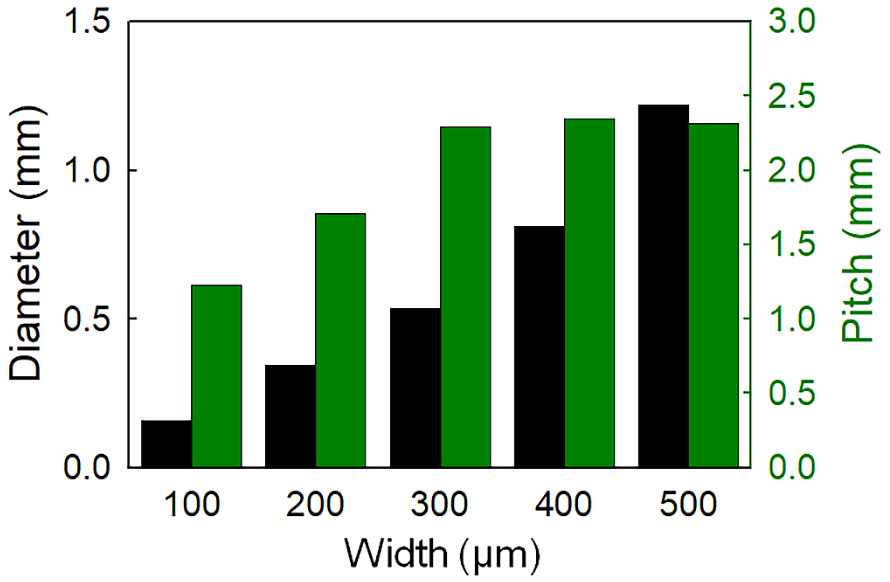

Figure S4. Rolling diameters and helix pitches of photo-structured azo-LCN helix structures. Sample length: 8 mm, azo-LCN strip thickness: 20 μm, light intensity: ~0.2 W cm^{-2} at 365 nm.

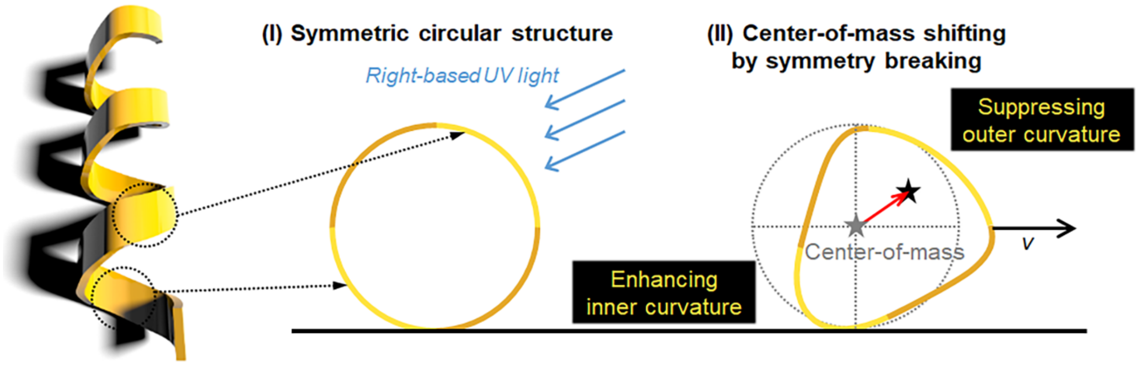

Figure S5. Rolling mechanism of spiral ribbon helix structure with monoclastic curvature, actuated by curvature-deformed symmetry breaking and resultant center-of-mass shifting under biased light irradiation.

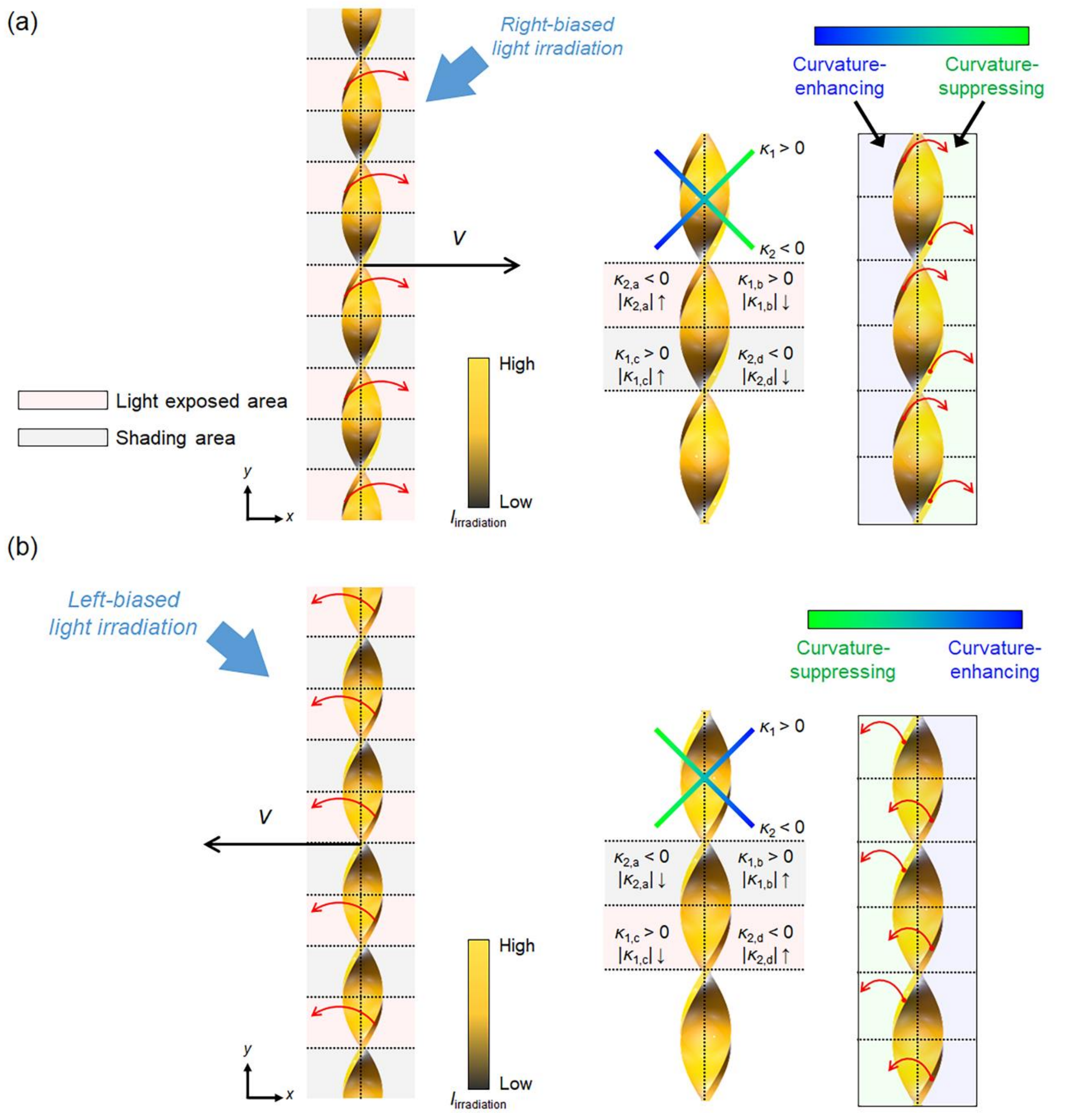

Figure S6. Rolling mechanism of helicoid helix structure with anticlastic curvature, actuated by curvature-deformed torsional helix-winding torque under (a) right-biased and (b) left-biased UV light irradiation. $\kappa_{2,a}$, $\kappa_{1,b}$, $\kappa_{1,c}$, and $\kappa_{2,d}$ are positional curvature conditions of κ_1 and κ_2, at each quadrant, constructing the anticlastic curvature shown in Figure S3.

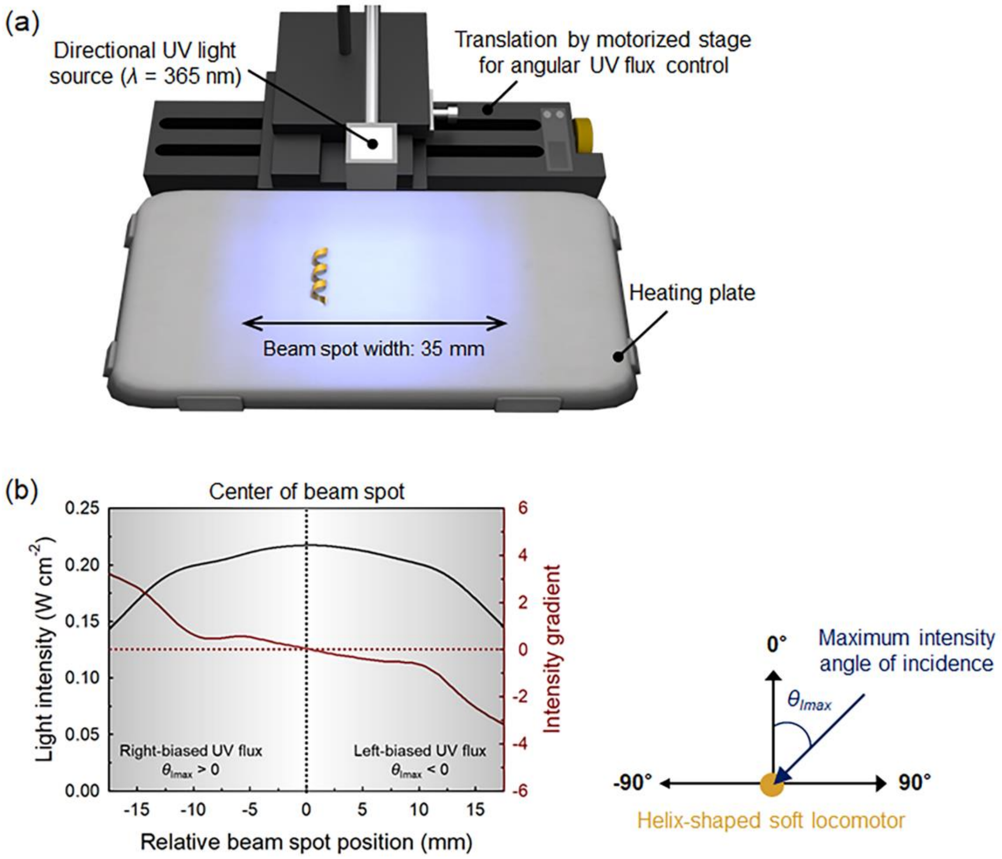

Figure S7. Experimental setup for photo-mechanical rolling. a) Schematic of the experimental setup for providing movable directional UV-light irradiation and elevated surface temperature conditions in photo-structuring, photo-actuated rolling, and photo-steerable rolling experiments. b) Horizontal light intensity and intensity gradient (W cm^{-3}) profile of directional UV light source. θ_{Imax} is the angle at which the incident light intensity is maximized and is zero at the center of the beam spot.

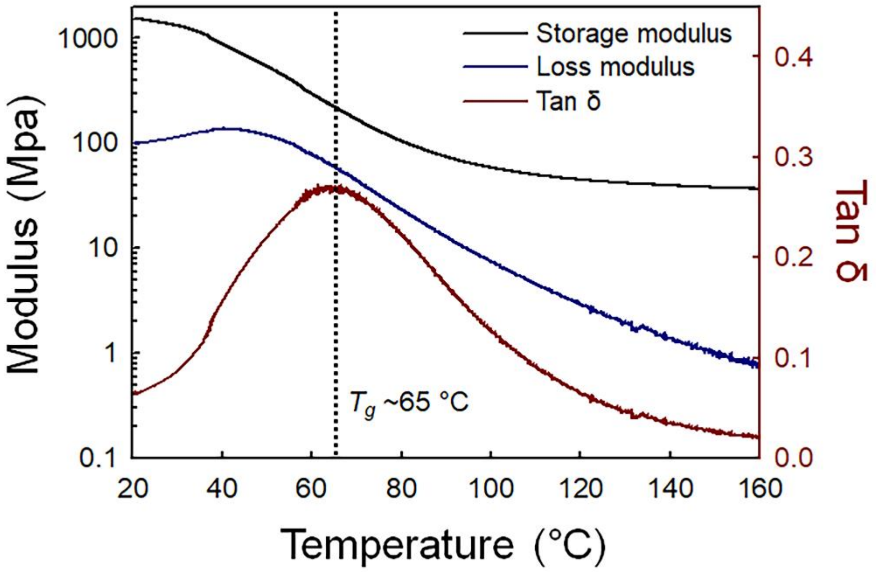

Figure S8. Temperature-dependent thermo-mechanical properties of an azo-LCN film.

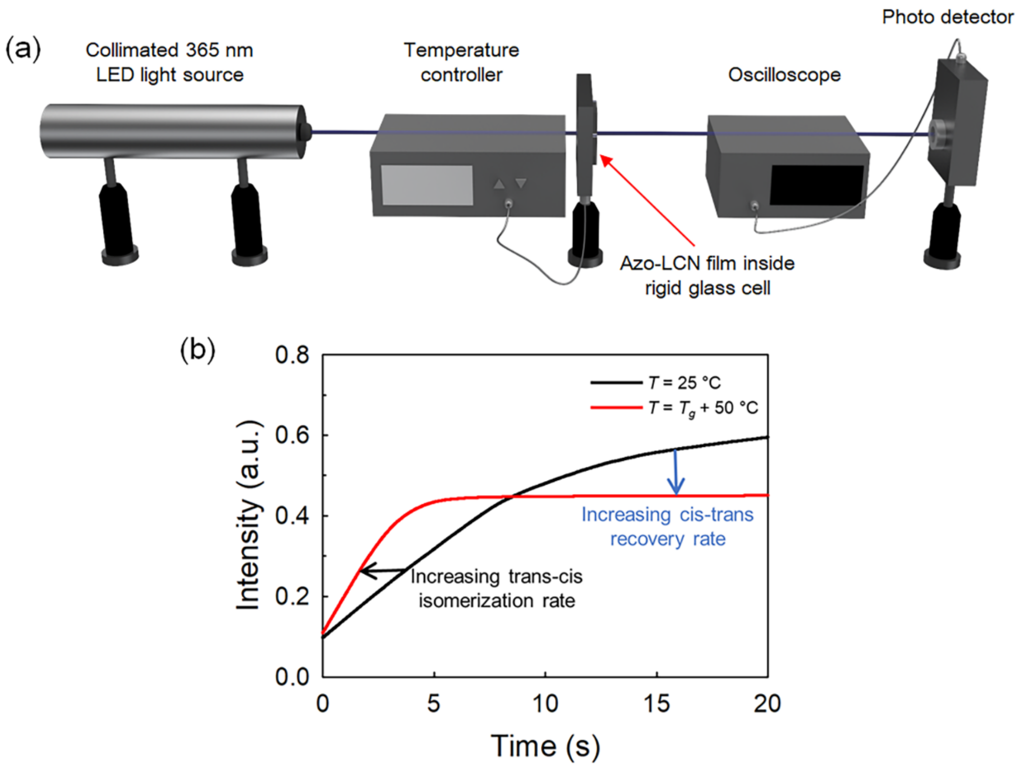

Figure S9. Temperature-dependent time-resolved UV transmittance of azo-LCN film. a) Schematics of the measurement setup. b) Temperature-dependent intensity variation with light irradiation time. Time constant values extracted by fitting curves are 8.85 s at 25 °C and 2.50 s at 115 °C. The thickness of the azo-LCN layer is confined between two rigid glass substrates: ~4 µm. Light intensity: ~0.05 W cm^{-2} at 365 nm.

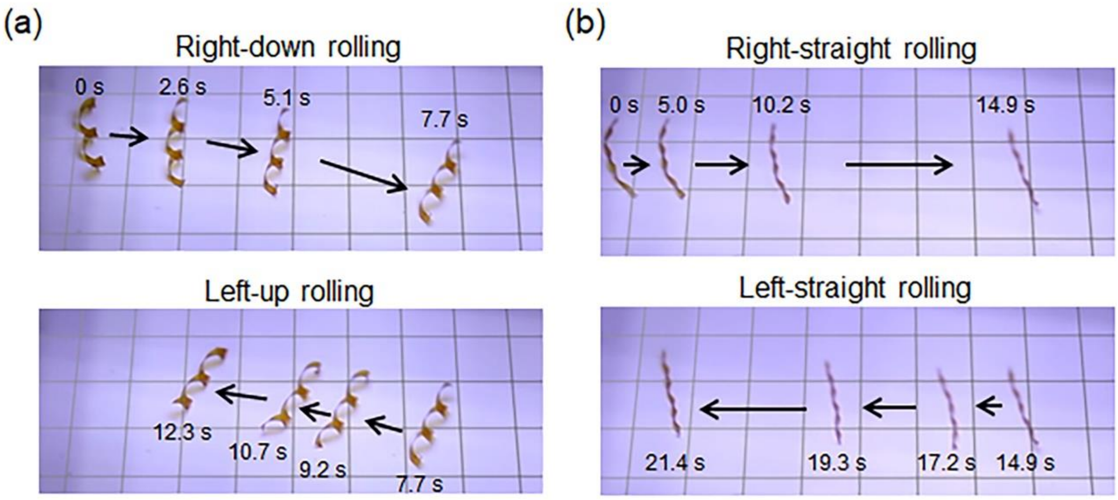

Figure S10. Round-trip rolling motions under a fixed-position light source with a spatially biased light-flux distribution. a) Biased rolling motions of spiral ribbon helix. b) Straight linear rolling motions of the helicoid helix. The film thickness of the azo-LCN strip was 20 μm before the photo-structuring. The laser-cut azo-LCN strip dimensions were 500 μm × 8 mm ($W \times L$) and 100 μm × 8 mm for spiral ribbon and helicoid helix azo-LCN rolling locomotors, respectively. For the horizontal light intensity profile, see Figure S7, Supporting Information. Grid length: 5 mm.

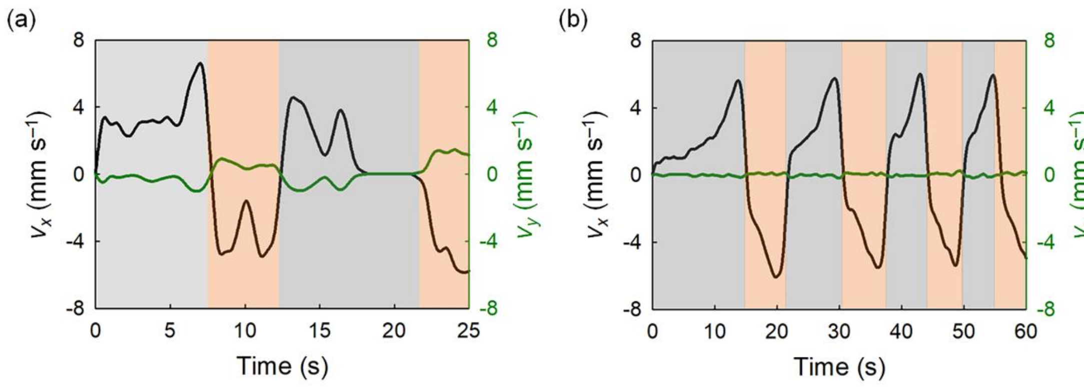

Figure S11. Photo-actuated rolling motilities of temporal v_x and v_y variations in repetitive round-trip motions. a) Spiral ribbon and b) helicoid helix rolling locomotors. The film thickness of the azo-LCN strip was 20 μm before the photo-structuring. The laser-cut azo-LCN strip dimensions were 500 μm × 8 mm ($W \times L$) and 100 μm × 8 mm for spiral ribbon and helicoid helix azo-LCN rolling locomotors, respectively. For the horizontal light-intensity profile, see Figure S7, Supporting Information. Gray and orange backgrounds in graphs correspond to right- and left-directional rolling motions, respectively.

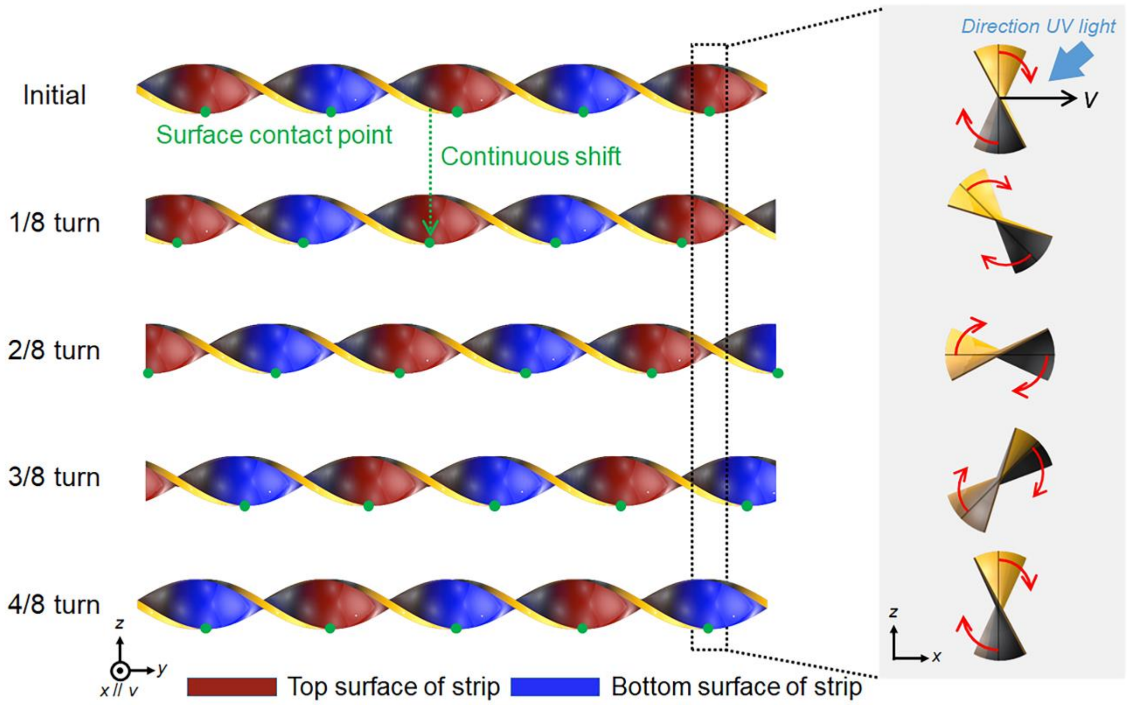

Figure S12. Helicoid helix structures with photo-induced rolling were observed in the *y*-*z* plane direction and the *x*-*z* plane direction. The helix axis is parallel to the *y* direction, and the substrate surface is parallel to the *x*-*y* plane. Periodic contact points with the substrate surface continuously shift along the helicoid helix's cross-sectional edge line. At any moment during the rolling process, when viewed from the same selected region along the helix axis, the cross-section of the helix appears to be rotating clockwise in both the light irradiation region and the light shadow region due to the curvature deformation.

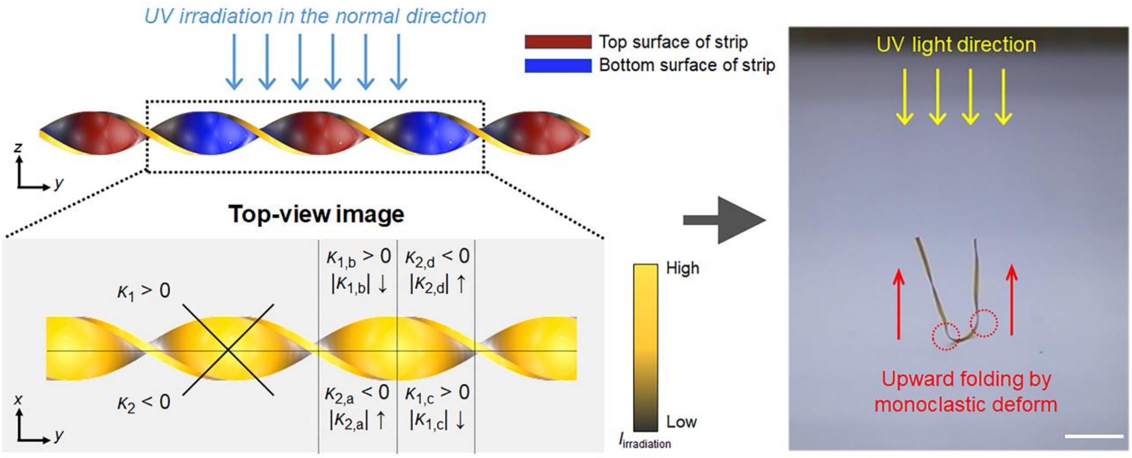

Figure S13. Positional curvature characteristics and their changes in the helicoid helix structure with the UV irradiation normally on the substrate and sample. Under the normal UV irradiation condition, monoclastic deformation occurs positionally randomly in helix nodes, resulting in photo-induced structural transition and subsequent upward folding with respect to the substrate surface, as shown in the image. Scale bar: 2 mm.

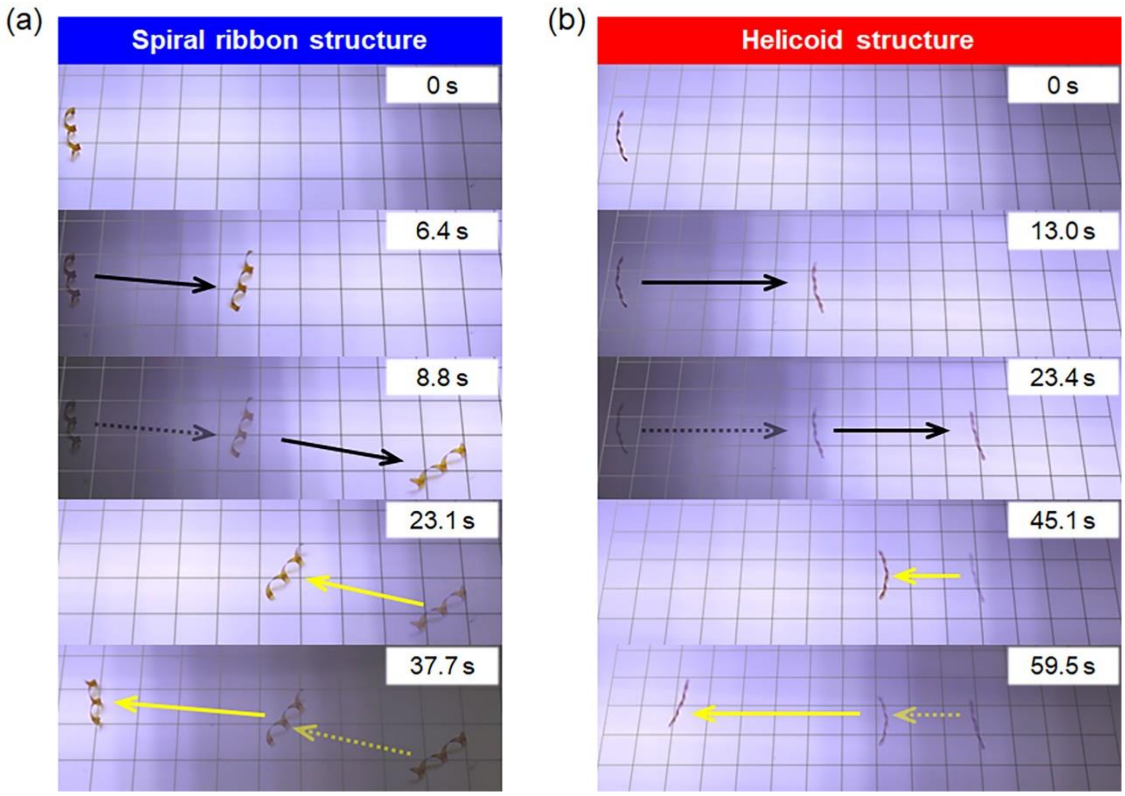

Figure S14. Photo-actuated rolling motilities of temporal v_x and v_y variations in repetitive round-trip motions. a) Spiral ribbon and b) helicoid helix rolling locomotors. The film thickness of the azo-LCN strip was 20 µm before the photo-structuring. The laser-cut azo-LCN strip dimensions were 500 µm × 8 mm ($W \times L$) and 100 µm × 8 mm for spiral ribbon and helicoid helix azo-LCN rolling locomotors, respectively. Gray and orange backgrounds in graphs correspond to right- and left-directional rolling motions, respectively.

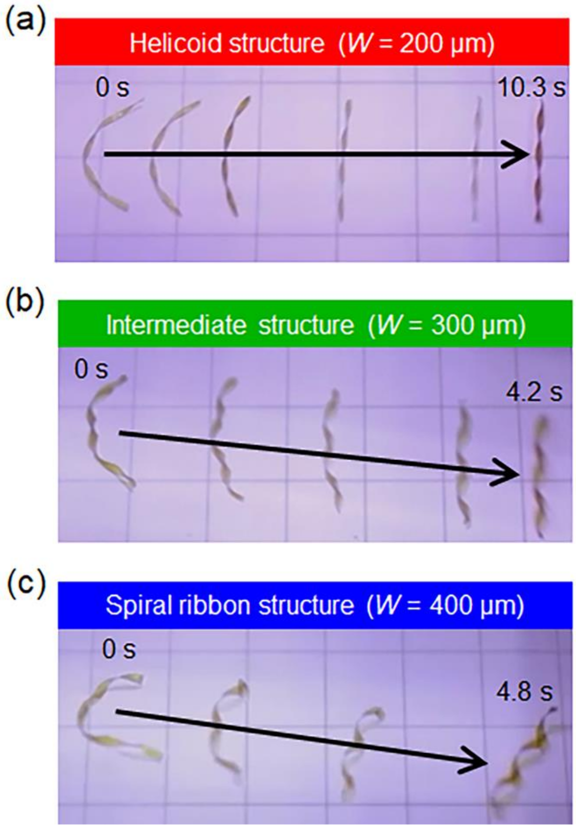

Figure S15. Photo-actuated rolling according to helix types controlled by the width-dependent aspect ratio of the azo-LCN strips. a) Straight linear rolling of the helicoid helix (*W* = 200 μm). b) Biased rolling of intermediate helix structure (*W* = 300 μm). c) Biased rolling of spiral ribbon helix (*W* = 400 μm). Grid length: 5 mm.

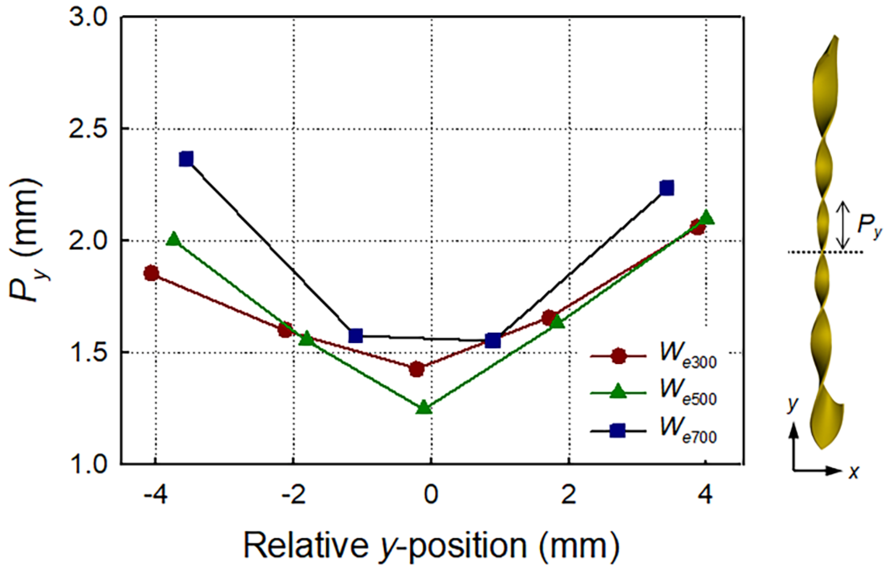

Figure S16. Positional pitch variation according to W_e in center-tapered helicoid locomotors.

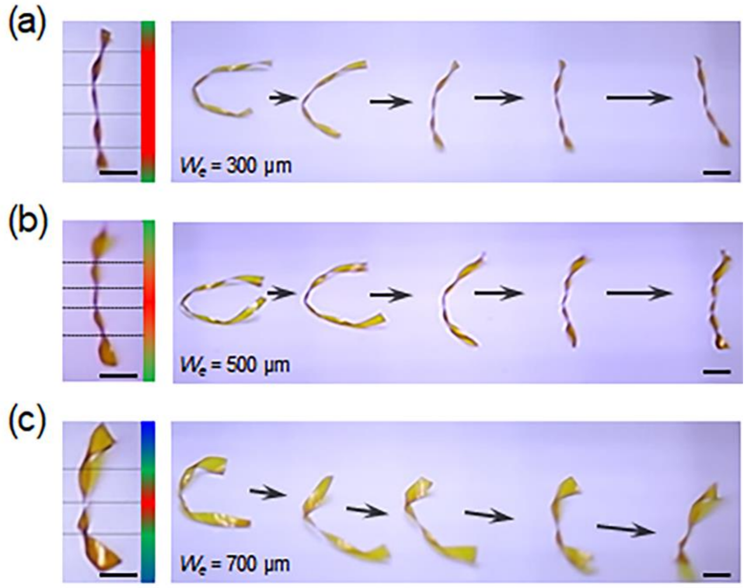

Figure S17. Photo-actuated rolling according to W_e of center-tapered helicoid locomotors. a) W_{e300}, b) W_{e500}, and c) W_{e700}. Scale bar: 2 mm.

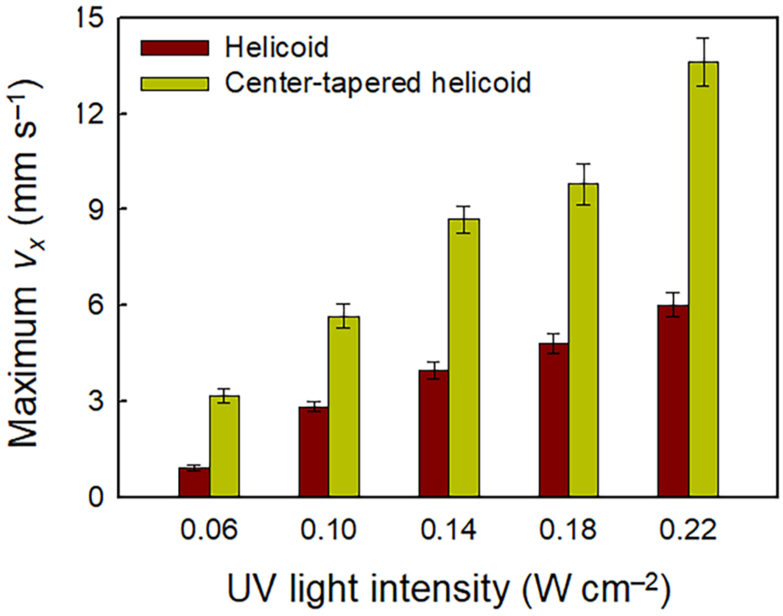

Figure S18. Maximum rolling velocity (v_x) of helicoid (W = 100 μm) and center-tapered helicoid (W_e = 500 μm) azo-LCN locomotors according to UV intensity. UV light intensity: maximum UV intensity value within the UV intensity profile, measured at the center of the beam spot (see Figure S7, Supporting Information) with varying UV intensity. Substrate temperature = 115°C.

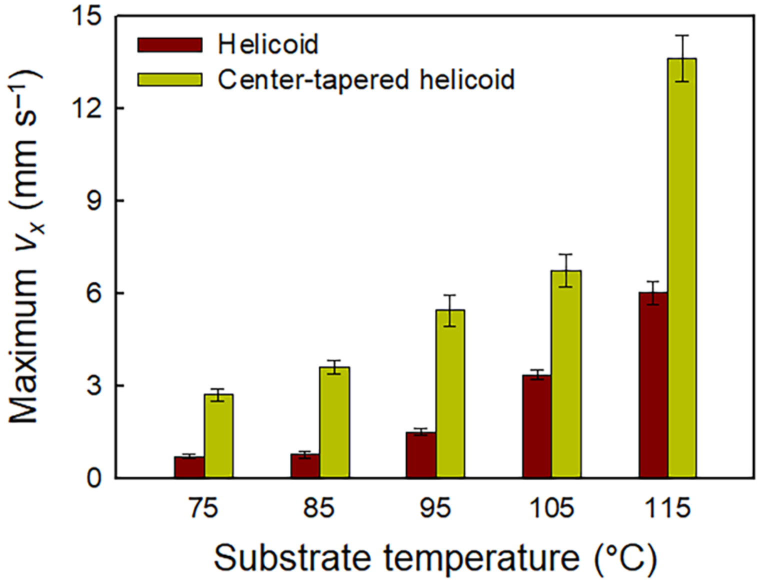

Figure S19. Photo-induced rolling motility measured by varying substrate temperature conditions: temperature-dependent maximum v_x of helicoid ($W = 100$ μm) and center-tapered helicoid ($W_e = 500$ μm) azo-LCN locomotors. For the horizontal light intensity profile, see Figure S7, Supporting Information.

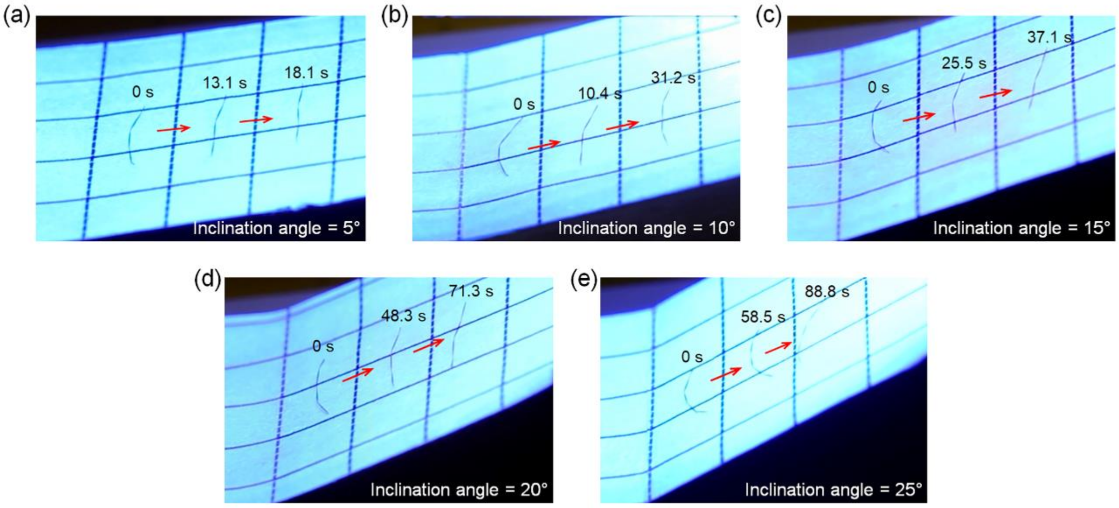

Figure S20. The slope-climbing motion of helicoid-structured rolling locomotors (W = 100 μm). Inclination angles of a) 5°, b) 10°, c) 15°, d) 20°, and e) 25°. Grid length: 5 mm.

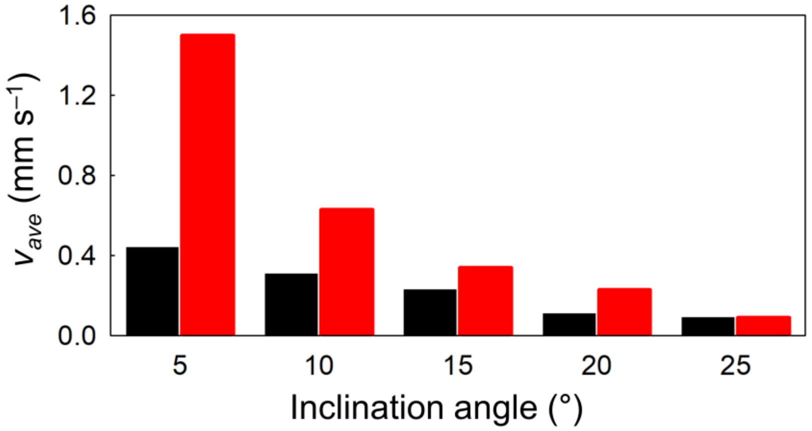

Figure S21. Photo-motilities are evaluated based on the average climbing velocity (v_{ave}) along the slopes according to the inclination angle. Black: helicoid rolling locomotor. Red: center-tapered helicoid rolling locomotor.

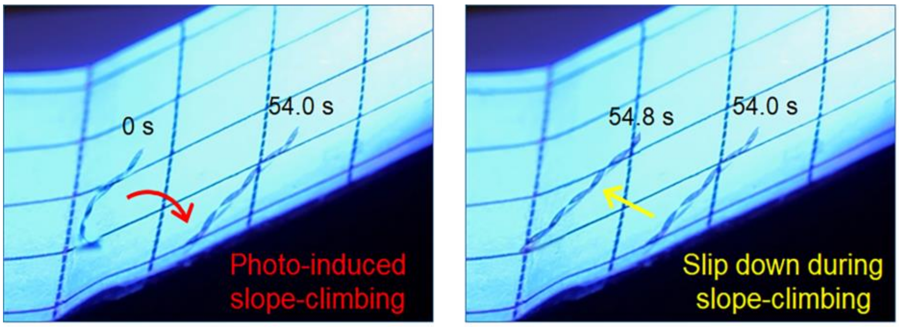

Figure S22. Snapshot images for climbing motion of an intermediate helix structure (*W* = 300 μm). Grid length: 5 mm, inclination angle: 25°.

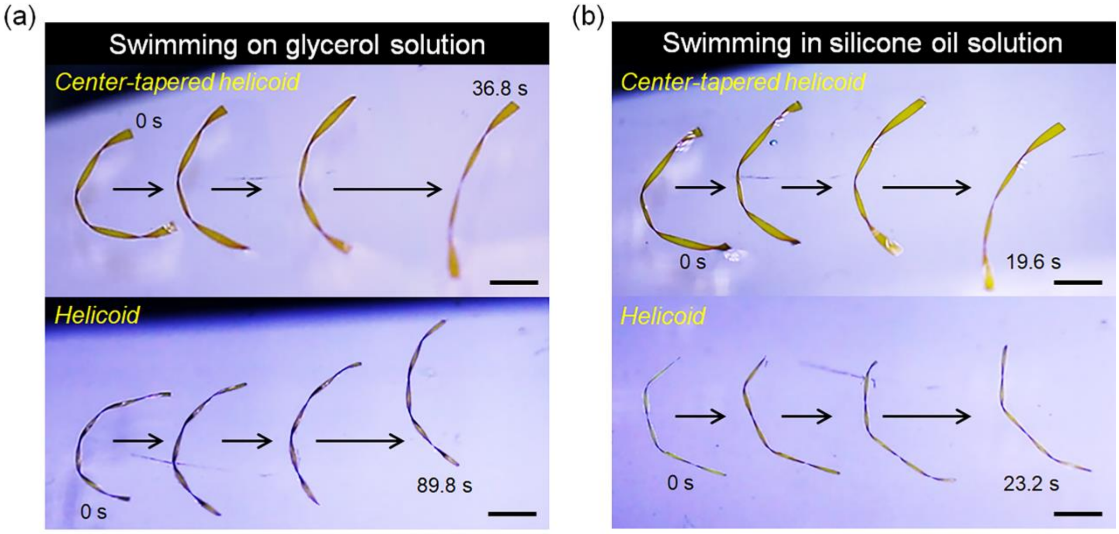

Figure S23. Snapshot images of the swimming motion of helicoid (W = 100 μm) and center-tapered helicoid (W_e = 500 μm) structures (a) on glycerol and (b) in silicone oil solutions. Scale bar: 2 mm.

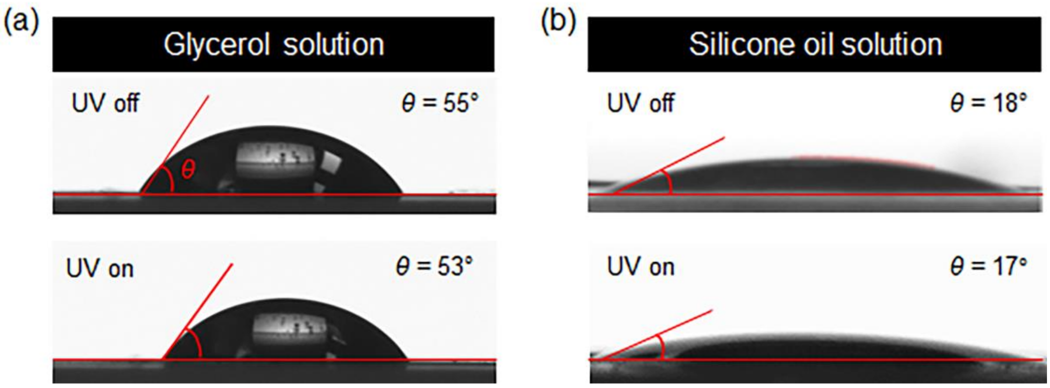

Figure S24. Contact angle measurement for (a) glycerol and (b) silicone oil solutions on azo-LCN film w/wo UV irradiation conditions.

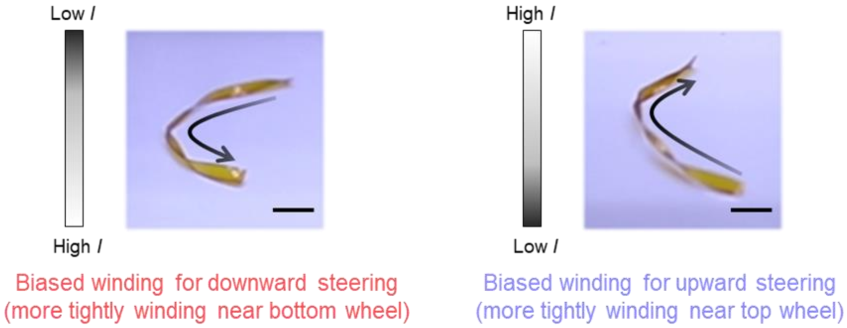

Figure S25. Snapshot images showing the initial step of photo-structuring with asymmetrically biased helix axis bending of center-tapered helicoid locomotor according to intensity bias of gradient light irradiation along the longitudinal direction of the azo-LCN strip. Scale bar: 3 mm.

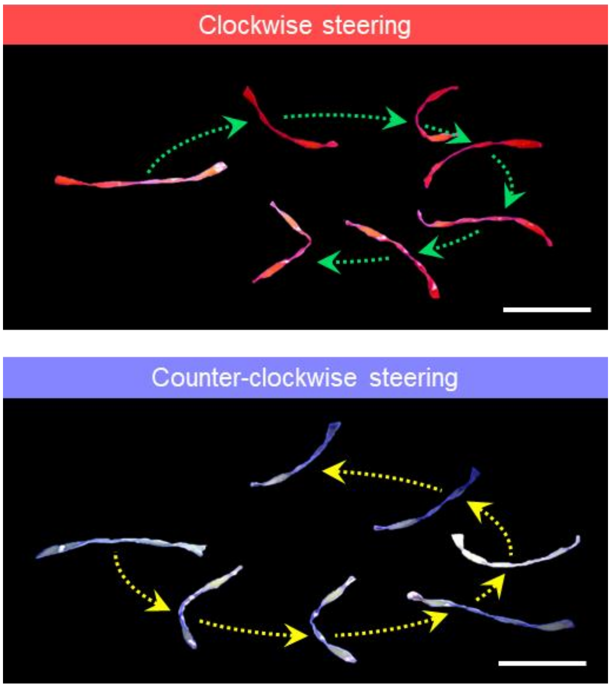

Figure S26. Clockwise and counter-clockwise photo-steered rolling motilities of center-tapered helicoid azo-LCN locomotor, controlled by moving actinic light. Scale bar: 5 mm.

Table S1. Moving modalities and motilities of photo-actuated rolling and crawling soft robots.

Reference	Moving modality	Effective body length (mm)	v_{max} (mm s^{-1})	RPM	On-demand steering
**This work [a]**	**Rolling (M, C, S)**	**0.63**	**13.63**	**413**	**O**
**This work [b]**	**Rolling (M, C, S)**	**0.16**	**6.03**	**720**	**X**
11	Rolling (M)	2.00	7.19	69	X
28	Rolling (M, C)	1.85	3.20	33	X
29	Rolling (M)	27.07	10.00	7	X
30	Rolling (M, C)	4.00	5.00	24	O
34	Rolling (M, C, S)	1.97	22.00	213	X
35	Rolling (M)	14.00	3.24	4	X
36	Rolling (M)	3.00	0.14	1	X
22	Crawling (M, C)	17.00	0.51	-	X
23	Crawling (M)	13.00	0.25	-	X
24	Crawling (M, C)	14.50	0.50	-	X
37	Crawling (M)	9.00	0.06	-	X
38	Crawling (M)	28.00	0.34	-	X
39	Crawling (M)	16.00	0.10	-	O
40	Crawling (M)	20.00	0.67	-	X

This work: [a] center-tapered helicoid structure (W_e = 500 μm), [b] helicoid structure (W = 100 μm). Moving modalities: moving on a flat rigid surface (M), climbing on the slope (C), and swimming in liquid (S). Effective body length: axial diameter for rolling motion and longitudinal length for crawling motion. v_{max} is the maximum velocity obtained when moving on a flat rigid surface.
